# Yeast transcription factor Msn2 binds to G4 DNA

**DOI:** 10.1093/nar/gkad684

**Published:** 2023-08-24

**Authors:** Duong Long Duy, Nayun Kim

**Affiliations:** Department of Microbiology and Molecular Genetics, University of Texas Health Science Center at Houston, Houston, TX 77030, USA; Department of Microbiology and Molecular Genetics, University of Texas Health Science Center at Houston, Houston, TX 77030, USA; MD Anderson Cancer Center UT Health Graduate School of Biomedical Sciences, Houston, TX 77030, USA

## Abstract

Sequences capable of forming quadruplex or G4 DNA are prevalent in the promoter regions. The transformation from canonical to non-canonical secondary structure apparently regulates transcription of a number of human genes. In the budding yeast *Saccharomyces cerevisiae*, we identified 37 genes with a G4 motif in the promoters including 20 genes that contain stress response element (STRE) overlapping a G4 motif. STRE is the binding site of stress response regulators Msn2 and Msn4, transcription factors belonging to the C2H2 zinc-finger protein family. We show here that Msn2 binds directly to the G4 DNA structure through its zinc-finger domain with a dissociation constant similar to that of STRE-binding and that, in a stress condition, Msn2 is enriched at G4 DNA-forming *loci* in the yeast genome. For a large fraction of genes with G4/STRE-containing promoters, treating with G4-ligands led to significant elevations in transcription levels. Such transcriptional elevation was greatly diminished in a *msn2Δ msn4Δ* background and was partly muted when the G4 motif was disrupted. Taken together, our data suggest that G4 DNA could be an alternative binding site of Msn2 in addition to STRE, and that G4 DNA formation could be an important element of transcriptional regulation in yeast.

## Graphical Abstract

**Graphical Abstract:**
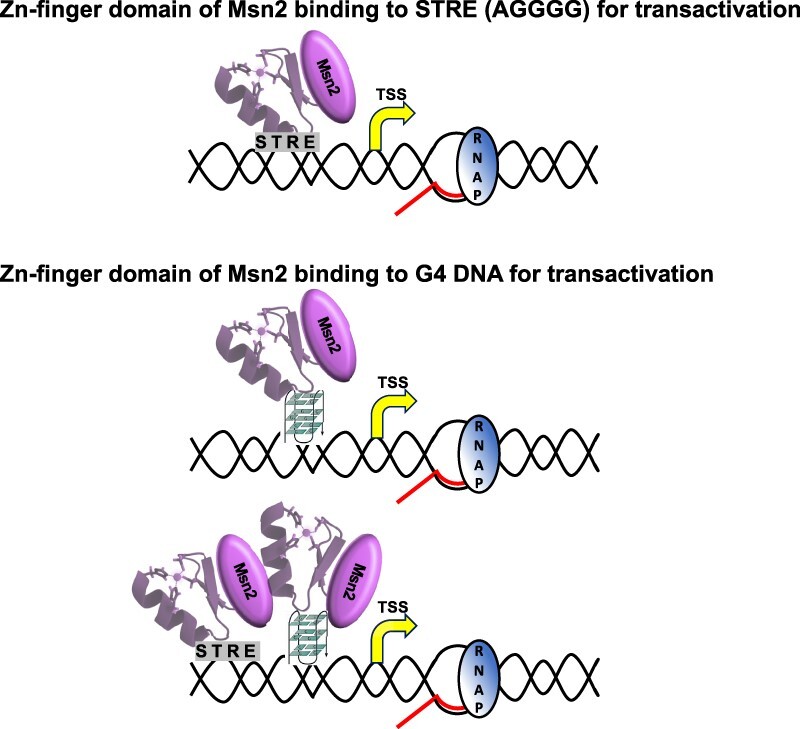

## INTRODUCTION

Guanine-rich DNA strands with the minimal consensus sequence of G_3_N_1–7_G_3_N_1–7_G_3_N_1–7_G_3_ can adopt a non-canonical structure called G4 quadruplex (G4 DNA). Four guanines interact together through Hoogsteen hydrogen bonds to form guanine tetrads which are stacked on top of each other and held by intervening loops to form the four-stranded G4 DNA (1–4). The G4 DNA structure formation *in vivo* has been confirmed by using specific anti-G4 antibodies, chemical biology, live cell imaging, and genomic technologies (1,3,5–7). To stabilize G4 structures, many small compound so-called G4 ligands have been developed, which bind selectively to G4 DNAs and have no or low binding to duplex DNA (8). There are 705 580 putative quadruplex-forming sequences (PQS) in the human genome. They are enriched mostly at the functional genomic regions including telomeres, immunoglobulin switching regions, ribosomal DNA, recombination hotspots, and gene promoters (9–12). More than 40% of human gene promoters harbor at least one G4 motif (12). G4-structure formation would affect cellular processes such as telomere maintenance, DNA replication, transcription, or induces genomic instability (1,11,13,14). Because of their potential biological functions and association with human diseases, G4 DNA is considered a promising therapeutic target (15–19).

The functional significance of G4 DNA at gene promoters was first characterized for the promoter of human *c-MYC* proto-oncogene where its formation reduces gene transcription (20,21). Subsequently, G4 formation in the promoters of other human proto-oncogenes, including *BCL2*, *c-KIT*, *c-MYB*, *HIF1α*, *KRAS*, *PDGF-A*, *VEGF*, etc. were shown to affect gene transcription (22–25). G4 DNA at the promoter can act as a facilitator or a repressor of transcription by recruiting or hindering the binding of transcription factors, respectively. For example, G4 at *c-MYC* promoter recruits CNBP and hnRNPK transcription factors to induce transcription but also recruits nucleolin, which occludes the binding of CNBP or hnRNPK to reduce *c-MYC* transcription (26,27). Interestingly, G4 DNA reportedly promotes the recruitment of gene-distal regulatory elements or enhancers through the dimerization of human G4 binding transcription factor YY1 (28).

The function of G4 DNA is largely dependent on G4 binding proteins. These proteins are often identified by biochemical experiments including affinity chromatography and mass spectrometry (29,30). Recently, there was a study using the yeast one-hybrid system with G4 DNA sequence placed in the promoter of a reporter gene to identify G4 binding proteins in yeast (31). The G4 binding proteins can be categorized into three main groups including G4 unwinding proteins, G4 stabilizing proteins, and G4 anchoring proteins (30). Human helicases such as WRN, BLM or FANCJ are the examples of G4 unwinding proteins that bind to and dissolve G4 structures. Nucleolin is an example of well-characterized G4 stabilizing proteins; while some transcription factors, DNA repair proteins, or chromatin remodeling proteins are G4 anchoring proteins that are recruited to and bind to G4 structures only (30,32).

G4-binding proteins often contains specific domains that recognize and bind to G4 quadruplex such as RGG/RG domain, RRM domain or OB-fold domain (30). However, there are emerging evidences pointing to zinc-finger proteins as the G4 DNA binders (32). The zinc-finger proteins are one of the largest protein families that are primarily considered transcription factors due to their DNA binding ability through zinc-finger domain (33,34). The zinc-finger structures are stabilized by the zinc ion which is bound by 4 amino acids in combination of cysteine and/or histidine residues. A zinc-finger protein can have from 1 to 40 zinc-finger domains that are connected through different linkers and each domain can have a different cysteine and histidine combination (35). Thus, zinc-finger proteins are very diverse in structures and classified into 30 different subgroups (36). But, in general, they can be categorized into 3 main subgroups including Cys2His2 (or C2H2), Cys4 and Zn2/Cys6 with C2H2 making up the most abundant subgroup with ∼1055 C2H2 proteins in human and ∼47 C2H2 proteins in budding yeast (35,36). Currently, four human zinc-finger proteins have been identified and well-charaterized as G4 binding proteins—CNBP, MAZ, SP1 and YY1 (32). Amongst them, MAZ, SP1 and YY1 are C2H2 zinc-finger protein whereas CNBP harboring C2HC motif and RG/RGG rich motif (32).

In addition to the works carried out in mammalian cell cultures, the budding yeast *Saccharomyces cerevisiae* has been a very useful model organism in studying the functional importance of G4 DNA. Early bioinformatics studies demonstrated the evolutionary conservation of the G4 DNA-forming sequences and their enrichment at specific genomic regions such as promoters (37). The effect of G4 ligands on the transcription, particularly for those genes with PQSs in their promoters, were also noted in the yeast (38). The genetic tractability of the yeast model system enabled uncovering of how G4 DNA-forming sequences contribute to elevated genome instability (39–41). Identification and/or characterization of novel G4-interacting proteins, including the highly conserved proteins such as Sub1, Top1, and nucleolin, were also carried out in the *S. cerevisiae* model system (29,42,43).

Here, we used the budding yeast as a model organism to further study how the G4 formation in gene promoters affects transcription. By using QGRS mapper, we have identified 37 yeast genes that have G4 motif in the promoter with 20 of them having G4 motif overlapping with stress response element (STRE), which is the binding site of a master regulator of stress response, Msn2. We show that Msn2 protein, a C2H2 zinc-finger transcription factor, binds to G4 DNAs *in vitro* with a binding affinity comparable to that reported for STRE and that Msn2-G4 interaction does not require STRE. In addition, Msn2 is enriched at G4 DNA-forming genomic sites *in vivo* and the transcription inductions of genes with promoter G4 by G4 ligand treatment is partially dependent on Msn2. Taken together, our data suggest that Msn2 transcription factor is a G4-binding protein and can regulate gene transcription through interaction with G4 DNA at gene promoters.

## MATERIALS AND METHODS

### Yeast strain and plasmid construction

Yeast strains used in this study were derived from YPH45 strain (*MATa*, *ura3-52 ade2-101 trp1*Δ*1*) (44) and are listed in Supplementary Table S1. The primers used for yeast strain and plasmid constructions are listed in Supplementary Table S2. Gene deletions were carried out by the standard one-step gene disruption method using loxP-flanked marker cassettes (45). The *MSN2-13Myc* strains were constructed as described in (46). The *SUB1-3xFlag* strain was constructed as described in (47). The *ATG39pG4Mut* and *TSL1pG4Mut* were introduced by the pop-in/pop-out two-step allele replacement method using *Spe*I-digested pRS306-ATG39pG4Mut-ATG39(1–200) and *Msc*I-digested pRS306-TSL1pG4Mut-TSL1(1–300) plasmids, respectively. These plasmids were constructed by introducing the G4 mutation into promoter sequence by PCR site directed mutagenesis method and then cloned into the integration plasmid pRS306 using NEB Builder HiFi DNA assembly master mix (NEB, Cat# E2621S). The sequence encoding for Msn2(596–704) zinc-finger domain (48) was amplified from yeast genome and cloned into plasmid pGEX4T1 using *Bam*HI and *Not*I sites for expressing GST-Msn2(596–704) in *Escherichia coli*. The plasmids are listed in Supplementary Table S3.

### RNA extraction, NanoString and qRT-PCR

Independent yeast colonies were inoculated in YEP-GE medium (1% yeast extract, 2% bacto peptone, 2% glycerol, 2% ethanol) and then grown overnight. Cells were diluted to OD_600_ ∼ 0.1 in YEP-GE medium and grown to mid-log phase (OD_600_ ∼ 0.5), and then further diluted to OD_600_ ∼ 0.1 in YEP-GE medium before adding 50 μM TMPyP4 (Sigma, Cat# 613560, prepared in water) or 25 μM PhenDC3 (Sigma, Cat# SML2298, prepared in DMSO). Cultures were grown until OD_600_ reached ∼0.5 and harvested by centrifugation. RNA was extracted using hot acid phenol method as described in (49). The extracted total RNAs were submitted to Genomic and RNA Profiling Core (GARP) of Baylor College of Medicine, Houston, Texas, USA for NanoString analysis using nCounter^®^ Custom CodeSet (NanoString Technologies, Inc.). The probe set included 37 genes (34 target genes and 3 house-keeping genes). The data were analyzed using nSolver 4.0 Analysis Software (NanoString Technologies, Inc.) and normalized to 3 house-keeping genes *ACT1*, *ALG9* and *TFC1* (50) which has high, medium, and low cellular copy numbers, respectively. *P*-value was determined by Student's *t*-test and *P* < 0.05 was considered significant. For RT-qPCR analysis, cDNA was synthesized using amfiRivert cDNA Synthesis Platinum Master Mix (GenDEPOT, Cat# R5600) and the cDNA copy numbers were determined by Bio-rad QX200 Droplet Digital PCR (ddPCR) system and QX200 ddPCR EvaGreen Supermix (Bio-Rad, Cat# 1864035). The ddPCR primers are listed in Supplementary Table S2. The mRNA fold change was calculated by normalizing to the mRNA copy number of control sample and to the mRNA copy number of the house-keeping gene *ALG9* (50). The Student's *t*-test was used to assess statistical differences with *P* < 0.05 considered significant.

### The oligo pull-down assay

*In vitro* DNA pull-down assays were performed as previously described in (42,47) with some modifications. Oligos with 3′ biotin-TEG attachment were purchased from Sigma and are listed in Supplementary Table S4. For each sample, 400 pmol of biotinylated oligos were folded in 100 μl buffer containing either 100 mM KCl or LiCl, and added to 100 μl of Streptavidin-Coupled M-280 Dynabeads (Invitrogen; Cat# 11205D). For yeast whole cell lysate preparation, cultures of yeast strain expressing either Msn2-13Myc or Sub1-3xFlag were grown and collected at an OD_600_ of 0.8–1.0. The western blotting analysis of oligo-bound and input samples (cell lysate) were carried out as described below.

### Protein purification and electromobility shift assay (EMSA)

The recombinant zinc-finger domain GST-Msn2(596–704) was expressed in *E. coli* BL21(DE3) cells harboring pGEX4T1-GST-Msn2(596–704) plasmid. Bacterial culture was grown in 500 ml LB medium supplemented with 100 μg/mL ampicillin and was incubated at 30°C with shaking at 150 rpm. Protein expression was induced with 0.05 mM IPTG (Thermo Fisher Scientific, Cat# 15529019) after the culture reached OD_600_ of ∼0.4 and then incubated with shaking at 150 rpm at 20°C for additional 1 hour before being harvested by centrifugation (4000 rpm, 4°C, for 15 min). Cell pellet was washed with cold PBS and stored at −80°C until further use. Frozen cell pellets were dissolved in lysis buffer (50 mM Tris–HCl pH 7.5, 500 mM NaCl, 10% glycerol, 5% Triton X-100, 100 μM ZnSO_4_, 5 mM DTT, 2 mM PMSF, Halt protease inhibitor (Sigma, Cat# 78430)) and disrupted by sonication (QSONICA sonicator). The crude extract (45 ml) was clarified by centrifugation (13 000 rpm, 4°C, for 15 min) before mixing with 3 mL glutathione agarose gel (Thermo Fisher Scientific, Cat# 16100) for batch purification. The unbound proteins were washed away using wash buffer (50 mM Tris–HCl pH 7.5, 500 mM NaCl, 10% glycerol, 0.05% Triton X-100, 100 μM ZnSO_4_, 1 mM DTT, 1mM PMSF, 0.1× Halt protease inhibitor, 20 mM MgCl_2_, 10 mM ATP (Sigma, Cat# A2383)) and the target protein was eluted using elution buffer (50 mM Tris–HCl pH 8.0, 100 μM ZnSO_4_, 1 mM DTT, 40 mM reduced glutathione (Sigma, Cat# G4251), 150 mM NaCl, 0.05% Triton X-100). The eluted protein was then exchanged to storage buffer (50 mM Tris–HCl pH 7.5, 50 μM ZnSO_4_, 1 mM DTT) using Zeba desalting spin column (Thermo Fisher Scientific, Cat# 89893) and then flash-frozen in liquid N_2_ and stored at −80°C for further use. The protein purity was analyzed by SDS-PAGE followed by Coomassie staining and protein concentration was determined by Total Protein Assay Kit (BioAssay System, Cat# QTPR-100). For EMSA, 5′ Cy5-labeled oligos (Sigma) (Supplementary Table S4) were resuspended in folding buffer (10 mM Tris–HCl pH 7.5, 100 mM KCl or 100 mM LiCl) and denatured at 95°C for 5 min before cooling down to room temperature overnight to allow G4 structures formation. Folded oligos (0.1 μM) were mixed with indicated amounts of purified GST-Msn2(509–704) in binding buffer (10 mM Tris–HCl pH 8.0, 1 mM DTT, 0.01% NP-40, 5% glycerol, 50 μM ZnSO_4_) and incubated at room temperature for 30 min before running on 6% nondenaturing TBE-polyacrylamide gel (Thermo Fisher Scientific, Cat# EC6365BOX) with 0.5× TBE buffer. Gel images were captured using Bio-Rad ChemiDoc™ MP imaging system and band intensity was measured by Bio-Rad Image Lab software. The signal intensities of bound and unbound fractions were determined and used for *K*_d_ calculation.

### Chromatin immunoprecipitation (ChIP)

ChIP of Msn2-13Myc protein was performed as described in (47,51) with some modifications. The mid-log phase cells at OD_600_ ∼ 0.1 were grown in synthetic complete (SC) media supplemented with 2% glucose until OD_600_ reached ∼0.5 and then shifted to SC media supplemented with 3% glycerol for 20 min to activate Msn2-13Myc protein (46). The chromatin DNAs were fragmented to ∼750 bp using Bioruptor Plus (Diagenode) with 7 cycles (30 s ON, 30 s OFF) at high amplitude. Each sample was incubated with 2.4 ng anti-cMyc monoclonal antibody (Thermo Fisher Scientific, clone 9E10, Cat# 13-2500) conjugated to 15 μl Protein G Dynabeads (Thermo Fisher Scientific, Cat# 10004D) overnight at 4°C. The primers used for ChIP-qPCR are listed in Supplementary Table S2. The relative DNA fold changes of target loci in ChIP samples were calculated by the ΔΔC_t_ method using ΔC_t_ value (IP – input) of target loci and ΔC_t_ value (IP – Input) of *UBC13* promoter which is not a target of Msn2 protein and does not contain G4 DNA motif. The relative DNA enrichments were calculated by dividing the relative DNA fold changes of tagged samples (containing Msn2-13Myc) to those of un-tagged samples. *P*-values were determined by Student's *t*-test and value that is less than 0.05 was considered significant.

### Western blotting

Protein samples were prepared as previously described in (52). Whole cell lysates were resuspended in 2× SDS sample buffer and boiled for 5 min at 95°C before loading onto 4–20% SDS-PAGE gels (Thermo Fisher Scientific, Cat# XP04202BOX). The proteins were then transferred to PVDF membrane using a Trans-Blot SD cell machine (Bio-Rad; Cat# 170-3940) and then incubated with anti-cMyc monoclonal antibody (Thermo Fisher Scientific, clone 9E10, Cat# 13-2500) followed by incubation with a secondary HRP-conjugated α-mouse Ig antibody (R&D Systems, Cat# HAF007). The membrane was treated with West-Q Femto ECL (GenDEPOT, Cat# W3680-010) and the signal was visualized using Bio-Rad ChemiDoc™ MP imaging system.

## RESULTS

### Screening for genes that have G4 motif in the promoter in *Saccharomyces cerevisiae*

In order to investigate the role of G4 DNA structures in transcription regulation, we searched for genes that have one or more G4 motifs in their promoters in the budding yeast *Saccharomyces cerevisiae*. We utilized QGRS mapper, a web-based G4 mapping tool (https://bioinformatics.ramapo.edu/QGRS/index.php) developed by the bioinformatics team of Ramapo College of New Jersey (53). The consensus G4 motif which we were screening for was G_≥3_N_≤10_G_≥3_N_≤10_G_≥3_N_≤10_G_≥3_ and the maximum length of the motif was 45 nt. Although it has been reported that the loop lengths of no longer than 7 nt is necessary to form very stable G4 structures (54), some studies have shown that longer loops can support G4 structures (55,56). But when the loop length exceeds 10 nt, the G4 folding rate is strongly reduced (57). Therefore, we chose 10 nt as the cutoff for the loop length in our screening. Since most of the yeast promoters are <1000 bp (58), any G4 motif located within 1000 bp upstream of gene start codon was considered in the gene promoter. Using QGRS mapper and the yeast genome sequences that were retrieved from the *Saccharomyces* Genome Database (SGD), we surveyed 6275 yeast genes to identify 37 genes containing G4 motifs in their promoters, with the G-scores ranging from 62 to 100 (Supplementary Table S5). These G4 motifs were located in either the sense strand or anti-sense strand of the promoters. We categorized these 37 genes into functional groups by their biological roles in Table 1. This list largely overlapped with the list of G4 DNA-forming genomic sites identified by Marsico et al. with 25 out of 37 genes containing G4 motifs verified through the G4-seq method (9).

**Table 1.** List of genes that have G4 motif in their promoter

Category	Genes
Autophagy	*ATG20, ATG39*
Cellular enzyme	*ADO1, ARE1, CAT2, CCA1, GPD1, HXK1, RAD54, RPH1, TRM3*
Membrane protein	*FIT2, GET2, PUN1*
Mitochondrial protein	*ATP10, COQ21, MRPL15*
Ribosomal protein	*RPL42B, RPS18A, RPS23A, YTM1*
Stress response	*HSP150, SOD1*
Transcription factor	*MSN4, RRN1, SUT1*
Trehalose synthesis	*TPS1, TSL1*
Others	*RSC6, SPG4*
Unknown function	*ART10, CUE4, ECM3, MEO1, YCR047W-A, YFL063W, YGR117C*

### Twenty genes have overlapping G4 motif and STRE in their promoters

Since G4 motifs contain guanine runs, we searched for transcription factors that have binding sites also containing guanine runs with at least three guanines. Amongst >150 transcription factors in budding yeast (59,60), we found 31 transcription factors that have a guanine run in their consensus binding sites (Supplementary Table S6). 74% of those transcription factors belong to zinc-finger family of transcription factors. In a previously published work, Kuang *et al.* used the H3K9ac modification, which correlates with transcription activity, to determine transcription factors enriched in stress condition and found 21 transcription factors all of which were zinc-finger proteins (61). We found that 67% of those transcription factors bind to the consensus binding sites that have guanine runs (Supplementary Table S6). This correlation indicates that the guanine runs in the promoter might play a role in stress response. We chose to first focus on zinc-finger transcription factors Msn2/4, the master regulators in stress response in budding yeast, which have the consensus binding site 5′-AGGGG referred to as ‘stress response element’ (STRE) (48,62–65). Among 37 genes with the promoter G4 motifs, we found 20 genes that also have one or more STREs overlapping with a G4 motif in the promoters (Table 2), including well-characterized Msn2/4 target genes such as *ATG39, HSP150*, *HXK1*, *SOD1*, *TPS1* and *TSL1* (46,61,64,66). We also categorized these 20 genes into biological groups as shown in Supplementary Table S7 such as autophagy genes (*ATG20*, *ATG39*), genes encoding for cellular enzymes (*ADO1*, *HXK1*, *RAD54*, *TRM3*), stress response genes (*HSP150*, *SOD1*), or trehalose synthesis genes (*TPS1*, *TSL1*). The colocation of G4 motifs and STREs in these gene promoters suggests that G4 DNA might regulate expression of these stress response genes.

**Table 2.** List of genes that have G4 motif overlapping with STRE in their promoter

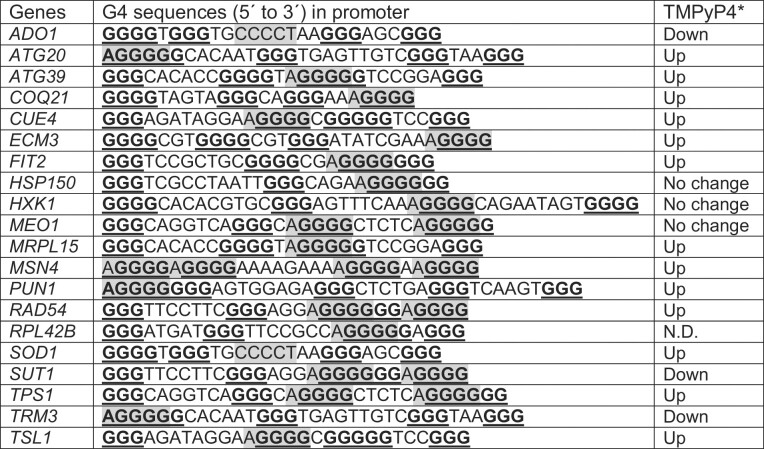

STREs (AGGGG or CCCCT) are highlighted in gray and guanine-runs making up a G4 motif are shown in bold. TMPyP4*—change in the transcript level upon treatment with 50 μM TMPyP4 according to the NanoString analysis (see Figure 6).

### Msn2 binds to G4 DNA structures *in vitro*

**Figure 1.**
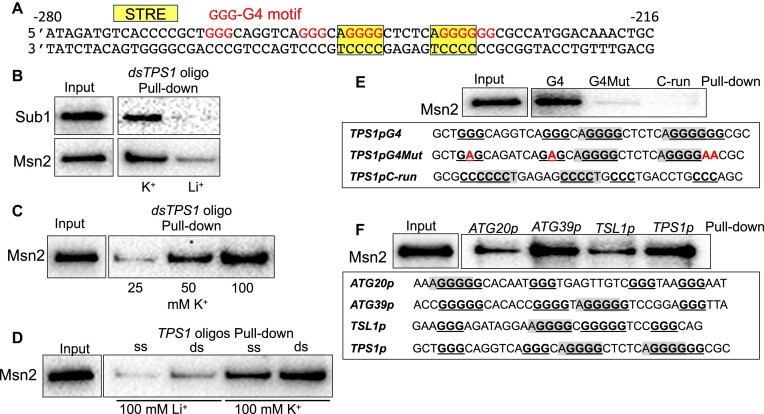
Msn2 protein binds to single- and double-strand oligos representing *TPS1* promoter sequence containing a G4 motif. (**A**) *TPS1* promoter sequence used for pull-down experiment with Msn2-13Myc. (**B**) *dsTPS1pG4* was formed in buffer containing either K^+^ or Li^+^ and used for pull-down experiments with either Sub1-3xFlag or Msn2-13Myc. (**C**) *dsTPS1pG4* was formed in buffer containing indicated K^+^ concentrations and used for pull-down experiments with Msn2-13Myc. (**D**) *dsTPS1G4* and single-stranded G-run containing *TPS1pG4* were formed in buffer containing either K^+^ or Li^+^ and used for pull-down experiments with Msn2-13Myc. **(E)** Msn2-13Myc pull-down experiment with single-strand oligos shown in the box below. Mutations introduced are shown in red. (**F**) Msn2-13Myc pull-down experiment with single-strand oligos shown in the box below. For (E) and (F), STREs are highlighted in gray. For all experiments, biotin-labeled oligonucleotides listed in the Supplementary Table S4 were used and Msn2-13Myc protein was detected by western blotting with anti-cMyc monoclonal antibody.

It is not clear how the binding of Msn2/4 transcription factors to STRE site is affected when G4 DNA forms in these promoters. We hypothesized that G4-formation would disrupt the binding of Msn2/4 to a STRE site since Msn2/4 have been reported to only bind to dsDNA with STRE (48,64). To test this hypothesis, we carried out the oligo pull-down assay with Msn2-13Myc protein using *TPS1pG4* oligos. *TPS1* is a well characterized target of Msn2 (61,66) and its promoter has 2 STRE sites overlapping a G4 motif (Figure 1A). First, we examined the binding of Msn2 protein to biotin-tagged *TSP1pG4* double-stranded oligos. We included 20 nt upstream and downstream of *TPS1p* G4 motif and annealed it to the oligo of complementary sequence in the presence of either 100 mM K^+^ or 100 mM Li^+^. As previously reported, K^+^ facilitates G4 formation while Li^+^ does not (4,67). The biotin-tagged folded oligos were then immobilized to magnetic beads and incubated with cell extract containing either Sub1-3xFlag protein or Msn2-13Myc protein. Sub1, a well-characterized G4 binding protein (29,47), served as a positive control. As expected, the binding of Sub1 to *dsTPS1pG4* was much more robust in the presence of K^+^ compared to that in the presence of Li^+^ (Figure 1B). Interestingly, in the presence of K^+^, Msn2-13Myc bound to *dsTPS1pG4* oligo more robustly than that in the presence of Li^+^ (Figure 1B) suggesting that G4 DNA formation facilitates the binding of Msn2 to this oligo. Since G4 formation is dependent on K^+^ concentration (4,67), we carried out the Msn2-13Myc pull-down assay with *dsTPS1pG4* oligos folded in various concentrations of K^+^ (Figure 1C). Increasing K^+^ concentration also increased the binding of Msn2-13Myc to *dsTPS1pG4* oligo indicating that G4 formation positively correlates with the binding of Msn2-13Myc.

We next asked whether Msn2 can bind to single-stranded (ss) *TPS1pG4* oligo. *ssTPS1pG4* oligo folded in either K^+^ buffer or Li^+^ buffer was used in the pull-down assay with Msn2-13Myc. Msn2-13Myc showed robust binding to *ssTPS1pG4* in the presence of K^+^ and much less pronounced binding in the presence of Li^+^ (Figure 1D). This data suggests that Msn2-13Myc binds to G4 structure forming from single-stranded DNA. To further confirm that Msn2-13Myc binds to G4 DNA structures, we did the pull-down with *ssTPS1pG4Mut* oligo, which could not form G4 structure but having 2 STRE sites intact, and *ssTPS1p-C-run* oligo, which was the reverse complement strand of *ssTPS1pG4* oligo (Figure 1E). Msn2-13Myc bound to *ssTPS1pG4* but did not bind to *ssTPS1pG4Mut* or *ssTPS1pC-run* indicating that Msn2-13Myc is binding specifically to the G4 DNA structure *in vitro*. Msn2-13Myc bound to G4 structures that formed from single strand oligo sequences from the promoters of other Msn2/4 target genes including autophagy genes *ATG20*, *ATG39*, and trehalose synthesis gene *TSL1 in vitro* (Figure 1F). Circular dichroism spectroscopy was used to determine the conformations of these oligos in presence of K^+^. *ATG20pG4*, *ATG39pG4* and *TSL1pG4* all showed maximum at 264 nm and minimum at 245 nm as typical of parallel G4 structures (68) whereas *TPS1pG4* appeared to have additional maximum at ∼295 nm suggesting a mixture of parallel and antiparallel form (Supplementary Figure S1).

### STRE is not required for the binding of Msn2 to G4 DNA *in vitro*

**Figure 2.**
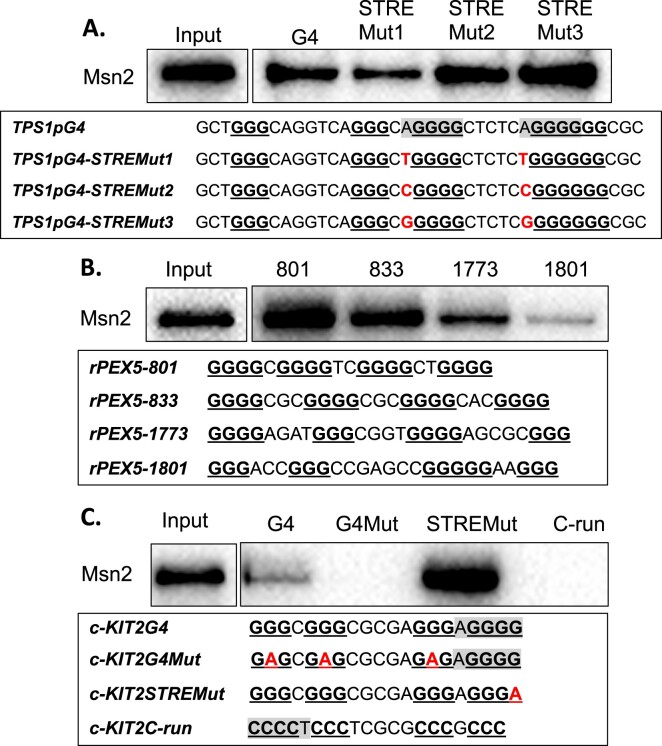
STRE is not required for the binding of Msn2 to G4 oligos. (**A**) Msn2-13Myc pull-down experiment with single-strand oligos shown in the box below with guanines within the G4 motif shown in bold. Mutations introduced are shown in red. (**B**) Pull down experiment with *rPEX5-801, rPEX5-833, rPEX5-1773* and *rPEX5-1801* oligos with Msn2-13Myc. Sequences are listed in the box below with guanines within the G4 motif shown in bold. (**C**) Pull-down experiment with single strand *c-KIT2G4*, *c-KIT2G4Mut*, *c-KIT2STREMut* and *c-KIT2C-run* with Msn2-13Myc. Sequences are listed in the box below with STREs highlighted in gray and guanine-runs within a G4 motif shown in bold. Mutations introduced are shown in red. For all experiments, biotin-labeled oligonucleotides listed in the Supplementary Table S4 were used and Msn2-13Myc protein was detected by western blotting with anti-cMyc monoclonal antibody.

The above G4 oligo pull-down data were carried out with oligos that have intact STREs overlapping the G4 motifs. We then examined whether Msn2-13Myc binds to G4 DNA-forming oligos is dependent on STRE. As shown in Figure 2A, mutating the two STRE sites on the *TPS1pG4* sequence did not significantly disrupt the binding of Msn2-13Myc to the oligos. And Msn2-13Myc bound to the non-STRE-containing *rPEX5* G4 oligos with sequences from rat *PEX5* gene (Figure 2B). Additionally, to confirm whether STRE is involved in the binding of Msn2-13Myc to a G4, we tested the *c-KIT2G4* oligo, which is a sequence derived from human *c-KIT2* gene and well-characterized to form a very stable G4 DNA structure (69). The pull-down data showed that Msn2-13Myc bound to the single-stranded *c-KIT2G4* oligo but did not bind to the *c-KIT2G4Mut* oligo or to the *c-KIT2C-run* oligo (Figure 2C). When the STRE was mutated, the c-*KIT2STREMut* oligo, was still bound by Msn2-13Myc confirming that STRE site is not required for G4 DNA binding of Msn2.

### Msn2 zinc-finger domain binds directly to G4 structures *in vitro*

**Figure 3.**
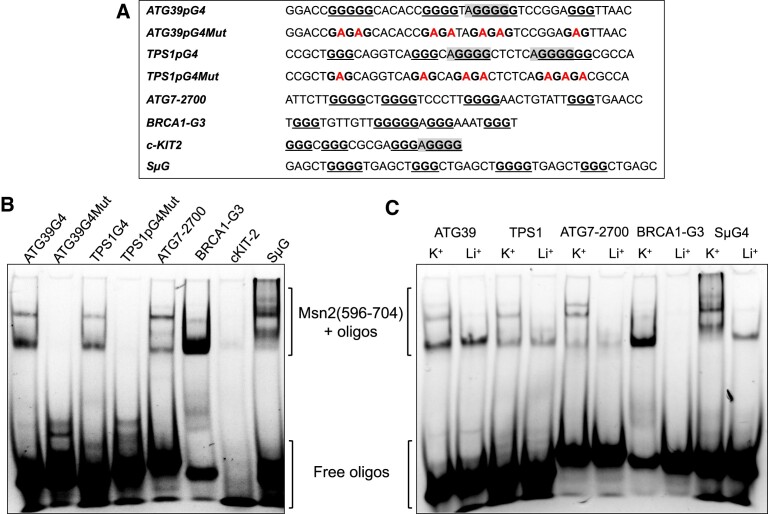
Purified Msn2 zinc-finger domain (A.A.596–704) binds directly to G4-forming oligos *in vitro*. (**A**) List of Cy5-labeled oligos used for EMSA experiment. STREs are highlighted in gray. Mutations introduced are shown in red. (**B**) Indicated oligos were folded in the presence of 100 mM K^+^ and subjected to EMSA with GST-Msn2(596–704). Free and high-mobility shifted bands are indicated in brackets. (**C**) Indicated oligos were folded in the presence of either 100 mM K^+^ or 100 mM Li^+^ as indicated above the lanes and subjected to EMSA with GST-Msn2(596–704). Free and high-mobility shifted bands are indicated in brackets. For all experiments, Cy5-labeled oligonucleotides listed in the Supplementary Table S4 were used.

In the pull-down experiments above, Msn2-13Myc could be binding to G4 DNA directly or indirectly through interaction with other G4 DNA binding factors. The Msn2/4 transcription factors belong to the zinc-finger DNA binding protein family and bind to double-stranded DNA at a STRE site through the zinc-finger domain (48,64). We hypothesized that Msn2 also binds to the G4 DNA structure through its zinc-finger domain. We cloned the sequence encoding for the Msn2 zinc-finger domain (A.A. 596–704) (48) into the plasmid pGEX-4T1 to express GST-Msn2(596–704) recombinant protein. We purified GST-Msn2(596–704) (Supplementary Figure S2) and confirmed that the purified protein binds selectively to double-stranded STRE site of *CTT1* promoter (Supplementary Figure S3A and B), a well-characterized target of Msn2 lacking G4 motif (46,61,64). The sequence specific binding of GST-Msn2(596–704) to *CTT1* promoter oligo was not affected by the presence of K^+^ versus Li^+^ (Supplementary Figure S3C). The purified GST-Msn2(596–704) protein bound directly to two G4 motif-containing oligos, *ATG39pG4* and *TPS1pG4* (Figure 3A and B, lanes 1 and 3) derived from the promoter sequences of yeast genes *ATG39* and *TPS1*, respectively. But GST-Msn2(596–704) did not bind when the G4 motifs were mutated as in *ATG39pG4Mut* and *TPS1pG4Mut* (Figure 3B, lanes 2 and 4). In order to show that the interaction between the G4 DNA and Msn2 is independent of the nature or origin of sequences, we also checked the direct binding of GST-Msn2(596–704) to other G4 motif-containing oligos. We took advantage of sequences that were previously reported to form stable G4 DNA. We found that GST-Msn2(596–704) bound directly to oligos derived from rat *ATG7* gene (70), rat *BRCA1* gene (71), and the mouse immunoglobulin switching region SμG (72) but not to an oligo derived from human *c-KIT2* gene (69) (Figure 3B). Notably, the Msn2 zinc-finger domain bound to G4 DNAs and often formed multiple band shifts suggesting that either there were more than one G4 conformation or the protein could bind to the G4 DNA with different molar ratios. To further confirm the direct, structure-specific binding of GST-Msn2(596–704) to G4 DNA, we also carried out EMSA with oligos that were folded in the presence of either 100 mM K^+^ or 100 mM Li^+^. The binding of GST-Msn2(696–704) was significantly more robust in the presence of K^+^ than that in the presence of Li^+^ (Figure 3C).

### Msn2 binds to G4 structures with an affinity similar to that of binding to STRE site

**Figure 4.**
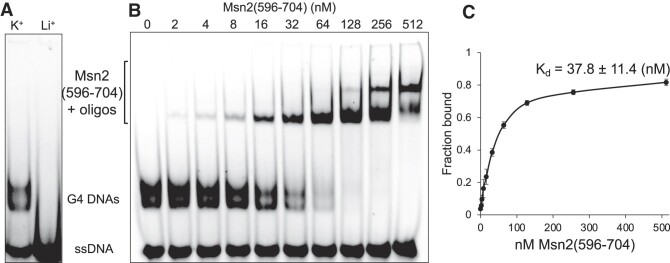
Determining the *K*_d_ of GST-Msn2(596–704) binding to *BRCA1-G3* oligo. (**A**) *BRCA1-G3* oligo folding in 100 mM K^+^ or 100 mM Li^+^ as indicated. G4 DNA with slow mobility appearing when folded in K^+^ is indicated at the left of the figure. (**B**) *BRCA1-G3* oligo was folded in 100 mM K^+^ and subjected to EMSA with indicated concentrations of GST-Msn2(596–704) protein. 1 pmol of *BRCA1-G3* oligo was used for each reaction. Unbound G4 DNA and G4-Msn2(596–704) bands are indicated. (**C**) EMSA-derived saturation binding curve of GST-Msn2(596–704) to *BRCA1-G3* G4 DNAs. Experiments were repeated three times and error bars indicating standard deviations are shown on graphs.

As previously reported, Msn2 binds to double strand STRE site with a dissociation constant (*K*_d_) of 23 ± 5.2 nM (48). We determined the *K*_d_ of Msn2 binding to G4 DNA using the *BRCA1-G3* oligo, which was confirmed to form parallel G4 structures according to the circular dichroism spectroscopy analysis (Supplementary Figure S1). As shown in Figure 3C, GST-Msn2(596–704) only bound to *BRCA1-G3* oligo that folded in K^+^ buffer but not in Li^+^ buffer. We also observed that *BRCA1-G3* oligo folded in K^+^ buffer showed a slower-migrating band, which appeared to be the G4 DNA structure (Figure 4A). For *K*_d_ calculation, a serial dilution of GST-Msn2(596–704) protein concentrations were used for the EMSA experiment with the *BRCA1-G3* oligo folded in K^+^ buffer. The EMSA data showed that the binding of GST-Msn2(596–704) to *BRCA1-G3* G4 DNAs increased with the increasing protein concentration and reached binding saturation at ∼100 nM (Figure 4B). The percentage of bound and unbound oligos were determined from band intensities, and the experiment was repeated 3 times to generate the binding curve for K_d_ calculation (Figure 4C). We determined that GST-Msn2(596–704) bind to *BRCA1-G3* G4 oligo with a dissociation constant of 37.8 ± 11.4 nM, which is similar to previously reported *K*_d_ of Msn2 to a STRE site. We repeated the experiment with the *ATG39pG4* oligo and determined the *K*_d_ of Msn2-interaction to be 30.2 ± 7.2 (nM) (Supplementary Figure S4). These results demonstrate that, in addition to a STRE site, G4 DNA structures could be an alternative binding site for the transcription factor Msn2.

### The Msn2-13Myc protein is enriched at G4 containing loci

**Figure 5.**
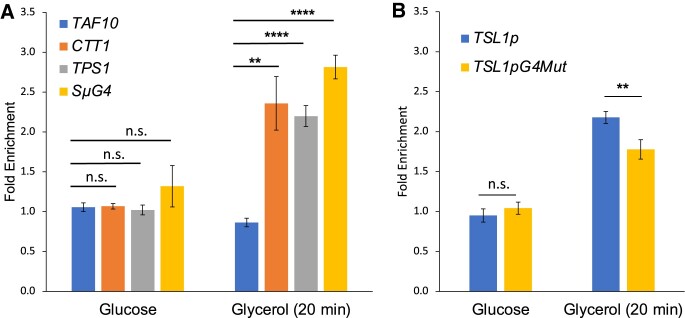
Msn2-13Myc enrichment at *TAF10, CTT1, TPS1*,*TSL1*, or *SμG4* locus. Yeast cultures either grown to mid-log phase in 2% glucose media or after a shift to 3% glycerol media for 20 min were harvested for ChIP with anti-cMyc monoclonal antibody. The Msn2-13Myc fold enrichments were determined by qPCR and Delta-Delta C_t_ method by normalizing to *UBC13* promoter DNA enrichment and to untagged samples. See Materials and Methods for technical details and Supplementary Table S2 for the sequences of primers used in qPCR. Sample size *N* = 4 and the significant difference was determined by Student's *t*-test. **P-value < 0.01, ****P-value < 0.0001 and n.s.; not significant. (**A**) Msn2-13Myc enrichment at *TAF10*, *CTT1, TPS1* and *SμG4*. (**B**) Msn2-13Myc enrichment at the *TSL1* promoter with a G4 motif (*TSL1pG4*; GGGAGATAGGAAGGGGCGGGGGTCCGGG) or with the G4-disrupting mutations (*TSL1pG4Mut*; GAGAGATAGGAAGGGGCGAGAGTCCGGG).

The surprising finding that Msn2 binds to the G4 DNA structures *in vitro* led us to investigate the binding of Msn2 to the G4 motifs *in vivo* by using the ChIP-qPCR approach. The promoter region of *CTT1* gene, which is a well-characterized target of Msn2/4 (66) and does not contain G4 motif, was chosen for the positive control. For the negative controls, the promoter regions of house-keeping gene*TAF10* with neither a G4 motif nor a STRE site were selected. Among the Msn2/4 targets that have G4 motif overlapping with STRE site, we focused on the *TPS1* promoter with two STRE sites (Table 2). In mid-log phase cells grown in 2% glucose containing media, there was no enrichment of Msn2-13Myc at *TAF10*, *CTT1* or *TPS1* promoters (Figure 5A). This result was expected since, when growing in rich media, Msn2 protein is largely excluded from nucleus (66,73). Therefore, we carried out the ChIP after the glucose down-shift to the glycerol-containing media, in which Msn2 is highly activated and triggers the transcription of stress response genes (46). After the glucose-to-glycerol down-shift, Msn2-13Myc was enriched at *pCTT1* and *pTPS1* by 2.36- and 2.2-fold, respectively, with no significant enrichment at the *TAF10* promoter. In order to determine whether Msn2-13Myc binds to the non-STRE-containing G4 site *in vivo*, we used a yeast strain harboring a 770-bp fragment of the mouse immunoglobulin switch region Mu (Sμ) containing 20 copies of the repeat (GAGCT)_n_GGGGT, which has been shown to form G4 DNAs *in vivo* (39,42,43,47,72,74). After the glucose-to-glycerol down-shift, Msn2-13Myc was enriched at *SμG G4* locus by ∼2.82-fold indicating that Msn2 localizes to G4 containing loci *in vivo* independent of STRE. We additionally showed that the enrichment Msn2-13Myc at another STRE/G4-containing promoter of *TSL1* gene was slightly but significantly reduced when the promoter sequence was mutated to no longer form a G4 DNA (Figure 5B,*TSL1pG4Mut*).

### The effect of G4 ligand on transcription of genes that have a G4 motif in the promoter

**Figure 6.**
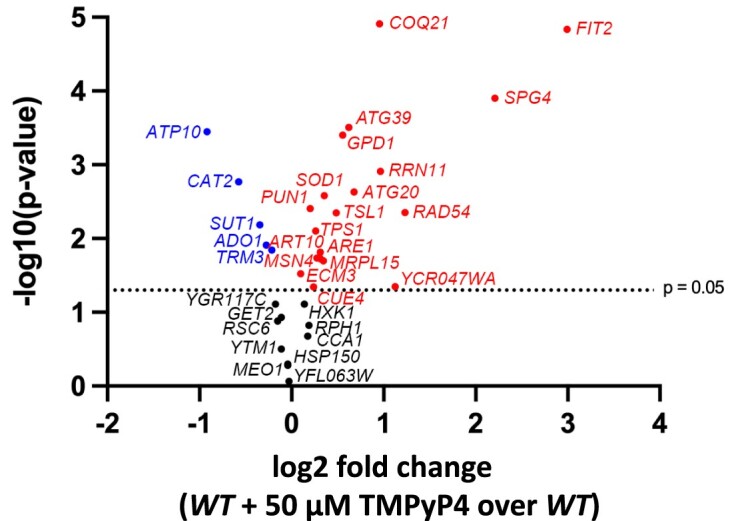
Effect of TMPyP4 on transcript level of genes with a G4 motif in their promoters. The mid-log phase WT cells were treated with 50 μM TMPyP4 for two generations and total RNAs were extracted for NanoString analysis with sample size *N* = 3 biological replicates. The transcript level of target genes was normalized to transcript levels of three house-keeping genes including *ACT1, ALG9*, and *TFC1*. X-axis represents mRNA fold change (TMPyP4 over untreated) while Y-axis represents *P*-value. The significant difference was determined by Student's *t*-test with *P*-value <0.05. Gene names with significant reduction or elevation in transcript levels in response to TMPyP4 are indicated in blue or red, respectively.

To address how the G4 DNA formation in the promoters affect gene transcription, we utilized TMPyP4, a G4 ligand that binds to and stabilizes G4 structures (75) and has been used in budding yeast (76). We measured the mRNA transcript levels of genes with G4 motifs in their promoters (Table 1) by using the NanoString technology (77). We treated mid-log phase WT cells with 50 μM TMPyP4 for two generations and then extracted the total RNA for NanoString analysis. After normalizing to transcript levels of three house-keeping genes *ACT1*, *ALG9* and *TFC1*, the data showed that stabilizing G4 structures by TMPyP4 upregulated the transcription of 19 genes, downregulated the transcription of 5 genes, and 10 genes had no change in the transcript levels (Figure 6). We excluded three ribosomal genes *RPL42B*, *RPS18A* and *RPS23A* from the NanoString analysis because they have close homologs. These data suggest that G4 formation in these promoters could affect transcription of these genes as either an enhancer or a suppressor. Because we had a small number of target genes and the effect of TMPyP4 on transcription differed, we could not identify any biological relevance amongst the upregulated or the downregulated genes.

Among the upregulated genes, we found *FIT2* gene encoding a cell membrane protein showed highest increase (8-fold) in the transcript level, while *ATP10* encoding for a mitochondrial protein was most reduced by TMPyP4 treatment (Figure 6). As previously reported, G4 DNA can bind to heme and act as a heme-buffer in human cells, and G4 ligand treatment leads to the release of heme from G4 DNAs, which eventually activates the transcription of iron regulon genes (78). Since the *FIT2* gene encodes for an iron transport facilitator (79), we speculated that the G4 ligand treatment affects the cellular heme level and activates the iron regulon in yeast cells. To confirm this, we measured mRNA level of *FET3* gene, which is another member of the iron regulon and encodes for a subunit of an iron permease complex and has no G4 motif in its promoter. As shown in Supplementary Figure S5, *FET3* mRNA level was also elevated when treated with TMPyP4, suggesting that the up-regulation of *FIT2* gene in the G4 ligand-treated cells is likely a result of perturbation in the cellular free heme levels and ensuing activation of transcription of the iron regulon genes.

### The loss of transcription factor Msn2/4 reduces *ATG20*, *ATG39*, *TPS1* and *TSL1* transcriptions induced by G4 ligand

**Figure 7.**
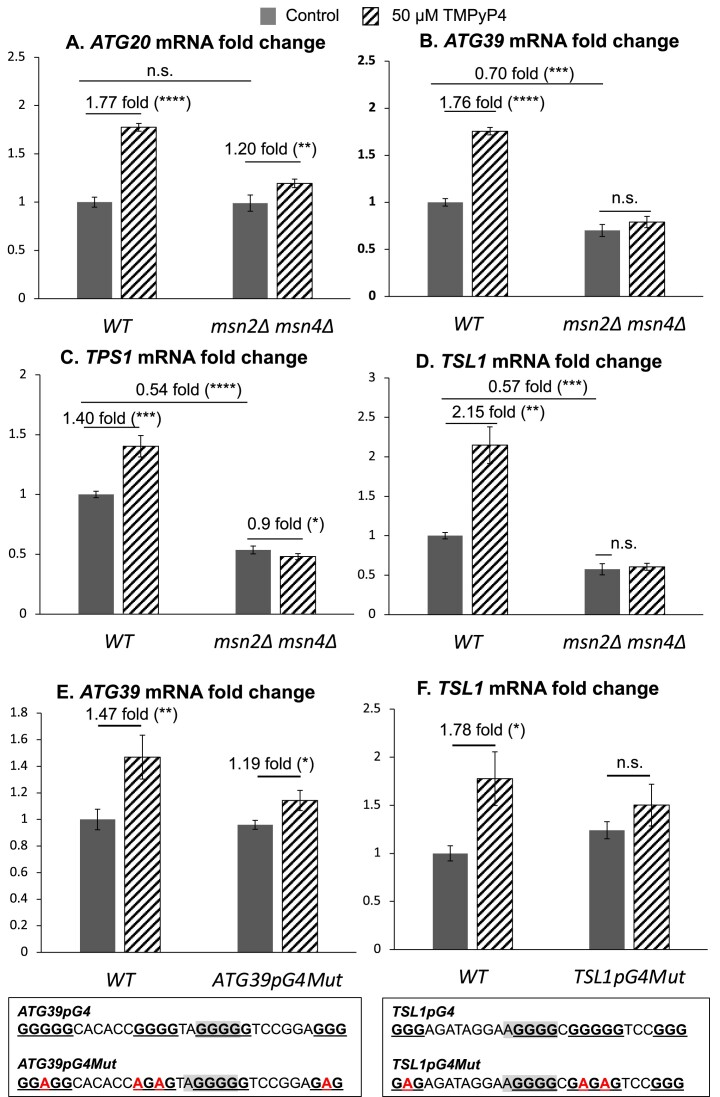
Deletion of *MSN2/4* reduces transcription induction caused by TMPyP4. Total RNAs were extracted for RT-qPCR analysis from the mid-log phase WT and *msn2*Δ *msn4*Δ cells treated with 50 μM TMPyP4 for two generations. qRT-PCR was used to measure the transcript level of each indicated genes. Primer sequences are listed in Supplementary Table S2. Sample size *N* = 4 biological replicates. The significant difference was determined by Student's *t*-test. **P-value < 0.01, ***P-value < 0.001, ****P-value < 0.0001, and (n.s.) not significant. The graphs show mRNA fold changes relative to that of untreated WT sample. The transcription levels in WT and *msn2Δ msn4Δ* were compared for (**A**) *ATG20*, (**B**) *ATG39*, (**C**) *TPS1* and (**D**) *TSL1*. For (**E**) and (**F**), mRNA fold change driven by *WT ATG39pG4* or *ATG39pG4Mut* (E) and *WT TSL1pG4* or *TSL1pG4Mut* (F) are shown. The mutated sequences are indicated in red in the boxes below each graph.

NanoString analysis showed that, among 20 genes with overlapping G4 and STRE, 13 genes including autophagy genes (*ATG20*, *ATG39*) and trehalose synthesis genes (*TPS1*, *TSL1*) were upregulated and three genes, *ADO1*, *SUT1* and *TRM3* were downregulated when treated with TMPyP4 (Table 2, Figure 6). To further test whether Msn2/4 regulates the transcription of these genes in the presence of G4 ligand, we deleted *MSN2* and *MSN4* genes and checked the level of G4 ligand-induced transcription for *ATG20*, *ATG39*, *TPS1* and *TSL1*, whose promoter G4s are bound by Msn2 (Figures 1 and 3). The loss of Msn2 and Msn4 resulted in significant decrease in the level of transcription even in the absence of TMPyP4 treatment for the genes *ATG39*, *TPS1*, and *TSL1* but not for *ATG20* (Figure 7A–D). Thus, it is possible that Msn2 or Msn4 is not involved in the transcription activation of *ATG20* in the conditions used here. The TMPyP4 treatment elevated the transcription of *ATG20*, *ATG39*, *TPS1*, and *TSL1* by 1.77-fold, 1.76-fold, 1.40-fold and 2.15-fold, respectively (Figure 7A–D). Upon the loss of Msn2 and Msn4, the transcription inductions by TMPyP4 for *ATG20* and*TPS1* genes were significantly reduced (Figure 7A and C). For *ATG39* and *TSL1* genes, the TMPyP4 treatment led to no significant increase in transcription in *msn2Δ msn4Δ* backgrounds (Figure 7B and D). Similar results were found when *WT* or *msn2Δ msn4Δ* cells were treated with a different G4 ligand, PhenDC3 (31,80–82) (Supplementary Figure S6). These data suggest that the transcription induction of these genes by a G4 ligand is partially Msn2/4 dependent. The deletion of Msn2/4 did not completely suppress the transcription induction caused by the G4 ligands for some of the genes indicating that there might be other transcription factors that contribute.

### G4 mutation reduces *ATG39* and *TSL1* transcriptions induced by G4 ligand

The formation of G4 DNA was previously reported to regulate transcription of human autophagy gene *ATG7* (70). We thus focused on the G4-dependent regulation of *ATG39*, one of genes with a PQS and STRE overlapping in the promoter. *ATG39* encodes a membrane receptor functioning in nucleophagy and ERphagy (83) and is a well characterized target of Msn2 (84). To assess the significance of G4 formation in transcriptional regulation of *ATG39*, we mutated the G4 motif in its promoter, which disrupted the G4 formation ability but maintained Msn2-recognition site STRE intact (Figure 7E). The transcription of *ATG39* with intact promoter sequence was upregulated when cells were treated with G4 ligands TMPyP4 or PhenDC3 (Figure 7B and Supplementary Figure S6B). When the G-runs within the G4 motif of *ATG39* promoter were mutated, the mRNA induction caused by the G4 ligand-treatment was moderately reduced (Figure 7E). We similarly introduced G4-disrupting mutations to the promoter of another Msn2 target gene, *TSL1*, encoding the regulatory large subunit of a trehalose 6-phosphate synthase/phosphatase complex, which plays important role in carbon storage for protecting the cells against environmental stress (61,85). In the absence of TMPyP4 treatment, mutating the *TSL1pG4* resulted in a moderately-elevated level of transcription, possibly due to the disruption of other transcription activator/repressor binding sites (Figure 7F). When treated with the G4 ligand TMPyP4, no significant elevation in transcription was observed for the *TSL1* gene with the G4-disrupting mutations in the promoter compared to the 1.78-fold elevation in transcription for the unmutated promoter. These data suggest that the formation of G4 DNA in the promoters of *ATG39* and *TSL1* genes facilitates gene transcription and confirms the involvement of G4 DNA structures in transcriptional regulation in budding yeast.

## DISCUSSION

The G4 DNA in gene promoters have been shown to regulate transcription of human genes (86). In this report, we focused on the G4 quadruplexes in gene promoters of budding yeast to get a better understanding of how the G4 formation could affect gene transcription. With the genomic guanine and cytosine (GC) content of 38.3% (SGD), we found only 0.6% of the *S. cerevisiae* genes (37 genes, Table 1 and Supplementary Table S5) having G4 motif in the promoter by using QGRS mapper, which is much less than that in human genome, whose GC content is 41% (87) but having 40% of promoter regions containing at least one G4 motif (12). These genes with the PQS-containing promoters were categorized into biological groups such as autophagy genes, cellular enzyme-coding genes, mitochondrial protein-coding genes, ribosomal protein-coding genes, stress response genes, transcription factor-coding genes, and trehalose synthesis genes (Table 1). Previously, Capra et al. identified 34 non-telomeric G4 motifs that are conserved in among 7 related yeast species allowing for the maximum loop length of 25 nt (37). In comparison, restricting to the maximum loop length of 10 nt, we identified 8 genes (*ATG39*, *CUE4*, *GPD1*, *HXK1*, *MEO1*, *MRPL15*, *TPS1* and *TSL1*) that contained conserved G4 motifs in the promoters (Table 1). This suggests that the G4 DNA in promoters of these genes may have important biological functions maintained through evolution. On the other hand, stabilizing the G4 DNA structures of those 37 genes by G4 ligands induced the transcription of 19 genes and reduced transcription of 5 genes (Figure 6) suggesting the role of G4 DNA in modulating transcription as a positive or a negative transcription regulator. We further confirmed the involvement of G4 in transcription by mutating the G4 motifs of *ATG39p* and *TSL1p*. Mutations disrupting the G4 motifs partly or completely offset the transactivating effect of G4 ligands for *ATG39pG4* or *TSL1pG4*, respectively (Figure 7E and F). Because there are many transcription factors that bind to the same promoter at different binding sites, examining the effect of G4 on transcription would be challenging. For example, any mutations intended to disrupt the G4 DNA formation could also affect the binding of other transcription factors. The *ATG39pG4* motif overlaps not only with the binding site of Msn2/4 transcription activator factors (STRE), but also with the binding site of Mig1/2 transcription repressor factors (84,88). Mutations we introduced to disrupt the *ATG39pG4* motif to generate *ATG39pG4Mut* maintained the STRE intact but disrupted the Mig1/2 binding site (Figure 7E). So alternatively, the disruption of Mig1/2 binding could be the reason why we observed the partial reduction in the G4 ligand-driven *ATG39* up-regulation.

The function of transcription factors depends on their binding to specific sequences in the gene promoters. Because G4 motifs contain guanine runs, we searched for transcription factors with known consensus binding sites that contain a guanine run and found 31 transcription factors (Supplementary Table S6). We focused on the transcription factor Msn2 because it is the master regulator of stress response and binds to the simple consensus STRE site, AGGGG (64–66). We found that 20 out of 37 G4-containing promoters have a G4 motif overlapping with a STRE (Table 2). This finding led us to question how G4 formation in these promoters affect the binding of Msn2 to a STRE site. Msn2 is a zinc-finger protein that was shown to bind to double-stranded DNA at STRE only (48,64). We initially hypothesized that the G4-formation would disrupt the binding of Msn2 to its consensus site. But, through an oligo pull-down technique using the yeast whole cell extract, we found that the G4 DNA formation did not interrupt but rather facilitated the binding of Msn2 to its target *dsTPS1pG4* oligo (Figure 1B and D). Furthermore, that binding was dependent on K^+^ and disrupted by Li^+^ (Figure 1B and C) suggesting the structure-specific binding of Msn2 to G4 formed by *dsTPS1pG4* oligo and stabilized by K^+^ (67). In further support of structure-specific binding of Msn2 to G4, Msn2 did not bind to the *ssTPS1C-run* oligo or the *ssTPS1pG4* oligo when G-runs are mutated (Figure 1E). Msn2 also bound to a number of other oligos containing both STRE and G4 motifs (Figure 1F). In order to show that STRE is not required for the binding of Msn2 to G4 DNAs *in vitro*, we demonstrated that Msn2 binds robustly to the *TPS1pG4* oligos with mutations disrupting the STRE site (Figure 2A). In addition, Msn2 strongly interacted with other G4-forming oligos, *rPEX5* and *c-KIT2*, indicating that Msn2 is a general G4 DNA-binding factor and not restricted to interacting with G4s present in the *S. cerevisiae* gene promoters (Figure 2B and C).

We have further evidence that Msn2 is a general G4 DNA-binding protein. First, for demonstration of direct interaction between Msn2 and G4 DNA, we showed that purified zinc-finger domain of Msn2 (A.A.596 to A.A.704) bound directly to a variety of G4-forming DNA oligos (Figure 3A). Similar to the full-length Msn2 in yeast whole cell extract, the interaction between Msn2(596–704) and G4 DNA is disrupted by mutations disrupting G4 motif but is promoted by the K^+^-containing buffer (Figure 3B and C). The apparent binding affinities were variable among different oligos and the specificity of binding remains to be resolved with further biochemical assays. Notably, *c-KIT2* oligo, which showed moderate but significant binding with full-length Msn2 when using yeast whole cell extract, did not bind to the purified Msn2(596–704). Repeating the binding assay with the *c-KIT2* oligo with additional flanking nucleotides did not result in significant interaction with the purified Msn2(596–704) (Supplementary Figure S7). For both *BRCA1-G3* and *ATG39pG4* oligos, the affinity of interaction with the Msn2(596–704), as inferred from the dissociation constants, were comparable to that of STRE-binding (Figure 4 and Supplementary Figure S4). Importantly, Msn2 protein was enriched at G4 DNA locus *SμG4 in vivo*, which was confirmed by ChIP experiment with Msn2-13Myc (Figure 5) demonstrating the *in vivo* interaction of Msn2 to G4 DNA. From these data, we are proposing that the G4 DNA structures in the promoters could be an alternative binding sites for Msn2 in addition to STRE site.

A recent study using the yeast one-hybrid system placed a G4 DNA motif in the promoter of a reporter gene as a bait to identify G4 DNA-binding proteins in budding yeast and found 157 proteins (31). However, Msn2 was not present in that list possibly because that screening was done in a normal growth condition in which Msn2 is largely localized in cytoplasm (66,73). In stress conditions, Msn2 is highly activated and sequence-specifically binds to the STRE to trigger transcription of stress response genes. Data presented here indicate that structure-specific binding of G4 DNA by Msn2 could be an additional element of such stress response and thus suggest that G4 DNA is a potential regulator of stress adaptation. There are additional evidences supporting this hypothesis. First, STREs overlapping with G4 motifs are present in the promoters of some stress response genes including autophagy genes (*ATG20*, *ATG39*), trehalose synthesis genes (*TPS1*, *TSL1*), *HSP150*, *HXK1*, *MSN4*, *RAD54*, and *SOD1* genes. Their transcription levels are significantly affected by G4 ligands (Figure 6). The loss of Msn2 and Msn4 reduced the transcription inductions of *ATG20*, *ATG39*, *TPS1* and *TSL1* caused by the G4 ligands (Figure 7 and Supplementary Figure S6). Also, a study had identified 21 transcription factors that were enriched at open chromatin in stress conditions (61), and 67% of those transcription factors including Msn2 have the binding sites containing guanine-runs (Supplementary Table S6). This includes Msn4, which is a highly similar homolog of Msn2 with their zinc-finger domains being 95% similar (89). We expect that the ability to bind to G4s is shared between Msn2 and Msn4. Furthermore, we also observed that Msn2 is enriched at the *SμG4* locus only upon glucose down-shift-to-glycerol media condition (Figure 5). As previously reported, the oxidation of guanine bases of G4 motifs in gene promoters under oxidative stress distorts the G4 structures triggering a change in gene transcription in human cells (90). More experiments in stress conditions would be needed in the future to get a better insight into the role of G4 DNA in the transcriptional regulation of stress response.

We did a literature search and found 31 transcription factors of budding yeast that have the binding sites containing a guanine-run (Supplementary Table S6) and, notably, 74% of them are zinc-finger proteins with 78% of these zinc-finger proteins being C2H2 type. The correlation between the guanine-run and C2H2 zinc-finger protein indicates a potential interplay between promoter-G4 quadruplex and the C2H2 subclass. Three of the four human zinc-finger proteins that have been reported to bind to G4 DNA (CNBP, MAZ, SP1 and YY1) are also C2H2 zinc-finger proteins (32). Furthermore, in a bioinformatics study of human, chimpanzee, mouse, and rat genomes, 9 transcription factors (AP-2, ETF, Kid3, KROX, MAZ, SP1, VDR, WT1, ZF5) have been identified to be enriched within 100 bp of conserved G4 motifs in gene promoters and 7 of them are zinc-finger proteins including 6 C2H2 proteins (91). In addition, a study has shown that an artificial C2H2 zinc-finger protein Gq1 binds selectively to telomeric G4 DNA (92,93). Taken together, these evidences suggest that C2H2 zinc-finger domain may be a new G4 DNA binding domain and our current data adds Msn2, another C2H2 subclass zinc-finger protein (64), to this growing list.

In summary, we have identified a new G4 binding protein, the C2H2 zinc-finger protein Msn2, and identified 37 yeast genes that have a PQS in the protomer with 20 of them having an overlapping STRE. We demonstrated that Msn2 binds to G4 DNA structures *in vitro* independent of a STRE with binding affinity similar to that of the STRE binding. We also confirmed that Msn2 protein is enriched at the G4-containing genomic loci under stress conditions and that Msn2 regulates the transcription of the promoter-G4 containing genes. These key findings suggest that G4 DNA may play a role in stress response through the interaction with the Msn2 transcription factor and that C2H2 zinc-finger proteins may be a new class of G4 binding protein.

## Supplementary Material

gkad684_Supplemental_File

## Data Availability

Raw data for NanoString Analysis were generated at Genomic and RNA Profiling Core at Baylor College of Medicine. Derived data supporting the findings of this study are available from the corresponding author [N.K.] on request. Other data supporting the findings of this study are either available within the article or its supplementary materials or will be made available from the corresponding author upon reasonable request.
