# Supplementary material for: Yeast transcription factor Msn2 binds to G4 DNA
Source: Nucleic Acids Res. 2023 Aug 24;51(18):9643–57. doi: 10.1093/nar/gkad684 (PMC10570036; doi:10.1093/nar/gkad684)
Supplement: gkad684_Supplemental_File [file gkad684_supplemental_file.pdf]

Supplemental Tables, Materials and Methods, Figures, and Legends

Table S1. The yeast strains used in this study

Strains	Relevant genotype	Source
WT	*MATa, ura3-52 ade2-101 trp1Δ1*	(1)
lys2SMUF	*MATa, ura3-52 ade2-101 trp1Δ1 his4Δ::lys2SMUF*	(2)
msn2Δ msn4Δ	*MATa, ura3-52 ade2-101 trp1Δ1 msn2Δ::loxP-Hyg^R-loxP msn4Δ::loxP-TRP1-loxP*	This study
MSN2-13Myc	*MATa, ura3-52 ade2-101 trp1Δ1 MSN2::MSN2–8xGLY-13MYC-TRP1 his4Δ::lys2SMUF*	This study
SUB1-3xFlag	*MATa, ura3-52 ade2-101 trp1Δ1 SUB1::SUB1-3xFLAG-URA3*	(3)
ATG39promG4Mut	*MATa, ura3-52 ade2-101 trp1Δ1 ATG39prom::ATG39promG4Mut*	This study
TSL1promG4Mut	*MATa, ura3-52 ade2-101 trp1Δ1 TSL1prom::TSL1promG4Mut*	This study
TSL1promG4Mut MSN2-13Myc	*MATa, ura3-52 ade2-101 trp1Δ1 TSL1prom::TSL1promG4Mut MSN2::MSN2-8XGLY-13MYC-TRP1*	This study

Table S2. Primers used for strain and plasmid constructions and qPCR in this study.

Primers	Sequences (5′ - 3′)	Used for
msn2KOF	TTTTTCAACTTTTATTGCTCATAGAAGAACTAGATCTAAAATGACGGTCGACCATGATTTCAATAGCGAACAGCTGAAGCTTCGTACG	Deletion of *MSN2*
msn2KOR	TTGAACAGAATTATCTTATTAGGAAAGATCTATCGAATTAAAAAAATGGGGTCTATTAAATGTCTCCATGAGGCCACTAGTGGATCTG	
msn4KOF	CCTTTATCAGTTCGGCTTTTTTTTCTTTTCTTCTTATTAAAAACAATATAATGCTAGTCTTCGGACCTAACAGCTTAAGCTTCGTACG	Deletion of *MSN4*
msn4KOR	AGAAAGAATATTATTTCTCCGAAAACTTGTCATACCGTAGCTTGTCTTGCTTTTATTTGCTTTTGACCTTAGGCCACTAGTGGATCTG	
MSN2-8xGly-13MycF	TAATTTGTCGCAACACATCAAGACTCATAAAAAACATGGAGACATTGGAGGTGGAGGTGGAGGTGGAGGTCGGATCCCCGGGTTAATTAA	Tagging 8xGly 13Myc to C-terminal of *MSN2*
MSN2-8xGly-13MycR	AAGCTTCATAAGTCATTGAACAGAATTATCTTATGAAGAAAGATCTATCGAATTAAAAAAATGGGGTCTAGAATTCGAGCTCGTTTAAAC	
pRS306-SacI-ATG39prom-1000UPF	ACTAAAGGGAACAAAAGCTGGGTACCCTGTAGTTTCAAAAACTGTGCTCC	Constructing plasmid pRS306-

pRS306-KpnI-ATG39-200R	ACTCACTATAGGGCGAATTGGAGCTCCGATTCATCTGTGCAGACTC	ATG39promG4Mut-ATG39(1-200)
ATG39promG4MutF	TGTATGGGTGGAGTTGACAGTTAAC**T**CTCCGGACCCCCTAC**T**C**T**GGTGTGCC**T**CCGGTCCATCTATCCATTTTGC	
ATG39prom-326UPR	GTTAACTGTCAACTCCACCCATAC	
pRS306-SacI-TSL1prom-500UPF	ACTAAAGGGAACAAAAGCTGGGTACCCTGTGTGTGTGGGAATGTATAAATG	Constructing plasmid pRS306-TSL1promG4Mut-TSL1(1-300)
pRS306-KpnI-TSL1-300R	ACTCACTATAGGGCGAATTGGAGCTCGTCCGAAGAGACAAGATCATC	
TSL1prom-214UPF	CAGCGGCTATGGTGTCAGTTACCTA	
TSL1promG4MutR	TAGGTAACTGACACCATAGCCGCTGCCCGGAC**T**C**T**CGCCCCTTCCTATCTC**T**CTTCGGC	
BamHI-MSN2-1789F	GCAGGATCCAATAATATAAGCAGTAGTAGTGTCAACTC	Constructing plasmid pGEX4T1-GST-Msn2(596-704)
NotI-MSN2-2115R	GCAGCGGCCGCTCTTAAATGTCTCCATGTTTTTTATGAGTCTTGATGTGTTGCG	
ALG9F	CACGGATAGTGGCTTTGGTGAACAATTAC	RT-qPCR
ALG9R	TATGATTATCTGGCAGCAGGAAAGAACTTGGG	
ATG20F	ACAGGTAGCGGTGGGAAATC	RT-qPCR
ATG20R	GCGGTATGCGGATCTGTTTC	
ATG39F	CAGAAACGCCGCTGGAAAAG	RT-qPCR
ATG39R	TCCCGACCAACATTGTTTTGC	

FET3F	GCCGATAACCCAGGTGTTTG	RT-qPCR
FET3R	CCTCCACGAGAACAAGACCC	
TPS1F	GAGATTCCTGACGATGAGAAGG	RT-qPCR
TPS1R	CTGAACCCGTTGTAGTGTAAGT	
TSL1F	CGATTCTTCTCCCCTTCTTCC	RT-qPCR
TSL1R	GGTCCTGCTGTTGCTGTTG	
CTT1promF	CCGAGAACTAAACTGCCTTCAC	qPCR for ChIP
CTT1promR	GTTCCTCATCATCTCTGGCTTC	
SμGF	GAGTAACCGGTGACGATGATATT	qPCR for ChIP
SμGR	CATTAAATGACCACGTTGGTTGA	
TAF10promF	ACCGCATTGAATCATTTGGTAG	qPCR for ChIP
TAF10promR	AGCATTGACAAGGAATCGAGAG	
TPS1promF	AAACTGCACTGAGGTTCTAAG	qPCR for ChIP
TPS1promR	ACTACCAAGTTAATCAATCTCTACC	
UBC13promF	CGCATCCGTATTGTTACCC	qPCR for ChIP
UBC13promR	TAGAGGTGTTTTGGTTCGTG	
TSL1promF	ATTTCTGCTCTTCTCGTCTCG	qPCR for ChIP
TSL1promR	GCAATCCCAAATACGCCATATC	

Table S3. Plasmids used in this study

Plasmids	Description	Source
pNK51	Replaced *KAN* marker of pUG6 plasmid from (4) with *HYG* marker, for deletion of *MSN2.*	Kim's Laboratory
pNK52	Replaced *KAN* marker of pUG6 plasmid from (4) with *TRP1* marker, for deletion of *MSN4.*	Kim's Laboratory
pRS306	Yeast integrative vector with a *URA3* marker and a MCS derived from pBLUESCRIPT, Amp^R	(1)
pGEX4T1	For generating GST tagged protein	Gift from Dr. Ji-Sook Hahn, Seoul National University, Korea.
pFA6A-13Myc-TRP1	For tagging 13Myc to *MSN2*	(5)
pRS306-ATG39promG4Mut-ATG39(1-200)	For generating *ATG39promG4Mut* yeast strain	This study
pRS306-TSL1promG4Mut-TSL1(1-300)	For generating *TSL1promG4Mut* yeast strain	This study
pGEX4T1-GST-MSN2(596-704)	For expressing GST-Msn2(596-704) in *E. coli*	This study

Table S4. Oligos used for pull-down assay and EMSA.

Oligos	Sequences (5′ - 3′)
TPS1pG4 (long sequence)	CTGATAGATGTCACCCCGCT**GGG**CAGGTCA**GGG**CA**GGGG**CTCTCA**GGGGGG**CGCCATGGACAAACTGCACT
TPS1pC-run-BtnTEG (long sequence)	AGTGCAGTTTGTCCATGGCGCCCCCCTGAGAGCCCCTGCCCTGACCTGCCCAGCGGGGTGACATCTATCAG-BtnTEG
TPS1pG4-BtnTEG	GCT**GGG**CAGGTCA**GGG**CA**GGGG**CTCTCA**GGGGGG**CGC-BtnTEG
TPS1pG4Mut-BtnTEG	GCT**GAG**CAGATCA**GAG**CA**GGGG**CTCTCA**GGGGAA**CGC-BtnTEG
TPS1pC-run	GCGCCCCCCTGAGAGCCCCTGCCCTGACCTGCCCAGC-BtnTEG
ATG20pG4-BtnTEG	AA**AGGGGG**CACAAT**GGG**TGAGTTGTC**GGG**TAA**GGG**AAT-BtnTEG
ATG39pG4-BtnTEG	ACC**GGGGG**CACACC**GGGG**TA**GGGGG**TCCGGA**GGG**TTA-BtnTEG
TSL1pG4-BtnTEG	GAA**GGG**AGATAGGAA**GGGG**C**GGGGG**TCC**GGG**CAG-BtnTEG
TPS1pG4-STREMut1-BtnTEG	GCT**GGG**CAGGTCA**GGG**CT**GGGG**CTCTCT**GGGGGG**CGC-BtnTEG
TPS1pG4-STREMut2-BtnTEG	GCT**GGG**CAGGTCA**GGG**CC**GGGG**CTCTCC**GGGGGG**CGC-BtnTEG
TPS1pG4-STREMut3-BtnTEG	GCT**GGG**CAGGTCA**GGG**CG**GGGG**CTCTCG**GGGGGG**CGC-BtnTEG

rPEX5G4-801-BtnTEG	**GGGG**C**GGGG**TC**GGGG**CT**GGGG**-BtnTEG
rPEX5G4-833-BtnTEG	**GGGG**CGC**GGGG**CGC**GGGG**CAC**GGGG**-BtnTEG
rPEX5G4-1773-BtnTEG	**GGGG**AGAT**GGG**CGGT**GGGG**AGCGC**GGG**-BtnTEG
rPEX5G4-1801-BtnTEG	**GGG**ACC**GGG**CCGAGCC**GGGGG**AA**GGG**-BtnTEG
c-KIT2G4-BtnTEG	**GGG**C**GGG**CGCGA**GGG**A**GGGG**-BtnTEG
c-KIT2G4Mut-BtnTEG	**GAG**C**GAG**CGCGA**GAG**A**GGGG**-BtnTEG
c-KIT2STREMut-BtnTEG	**GGG**C**GGG**CGCGA**GGG**A**GGGA**-BtnTEG
cKIT2C-run-BtnTEG	CCCCTCCCTCGCGCCCGCCC-BtnTEG
Cy5-CTT1p (sense strand)	Cy5-CTCTTTTTCAAGGGGATCACCGGTAAGGGGCCAATCTGTG
CTT1p (anti-sense strand)	CACAGATTGGCCCCTTACCGGTGATCCCCTTGAAAAAGAG
Cy5-CTT1pSTREMut (sense strand)	Cy5-CTCTTTTTCATGTGTATCACCGGTATGTGTCCAATCTGTG
CTT1pSTREMut (anti-sense strand)	CACAGATTGGACACATACCGGTGATACACATGAAAAAGAG
Cy5-ATG39pG4	Cy5-GGACC**GGGGG**CACACC**GGGG**TA**GGGGG**TCCGGA**GGG**TTAAC

Cy5-ATG39pG4Mut	Cy5-GGACC**GAGAG**CACACC**GAGA**TA**GAGAG**TCCGGA**GAG**TTAAC
Cy5-TPS1pG4	Cy5-CCGCT**GGG**CAGGTCA**GGG**CA**GGGG**CTCTCA**GGGGGG**CGCCA
Cy5-TPS1pG4Mut	Cy5-CCGCT**GAG**CAGGTCA**GAG**CA**GAGA**CTCTCA**GAGAGA**CGCCA
Cy5-ATG7-2700	Cy5-ATTCTT**GGGG**CT**GGGG**TCCCTT**GGGG**AACTGTATT**GGG**TGAACC
Cy5-BRCA1-G3	Cy5-T**GGG**TGTTGTT**GGGGG**A**GGG**AAAT**GGG**T
Cy5-c-KIT2	Cy5-**GGG**C**GGG**CGCGA**GGG**A**GGGG**
Cy5-SμG	Cy5-GAGCT**GGGG**TGAGCT**GGG**CTGAGCT**GGGG**TGAGCT**GGG**CTGAGCT

Table S5. List of genes that have G4 motif in the promoter found by using QGRS mapper

Name	G4 sequence	Location in promoter	Strand	G-Score	GO Term
ADO1 / YJR105W	**GGGG**T**GGG**TGCCCCTAA**GGG**AGC**GGG**	-383 to -358	Sense	65	Purine nucleobase metabolism
ARE1 / YCR048W	**GGG**CACC**GGG**ATACCGTTCC**GGG**C**GGG**	-709 to -682	Antisense	63	Sterol metabolism
ART10 / YLR392C	**GGG**TTGA**GGG**TGAGAGA**GGG**TCCT**GGG**	-283 to -256	Sense	69	N.D.
ATG20 / YDL113C	**GGG**CACAAT**GGG**TGAGTTGTC**GGG**TAA**GGG**	-169 to -139	Antisense	66	Autophagy
ATG39 / YLR312C	**GGG**CACACC**GGGG**TA**GGGGG**TCCGGA**GGG**	-326 to -297	Antisense	71	Autophagy
ATP10 / YLR393W	**GGG**TTGA**GGG**TGAGAGA**GGG**TCCT**GGG**	-517 to -490	Antisense	69	Mitochondrial ATP synthase complex assembly
CAT2 / YML042W	**GGG**C**GGGGGG**AAGTGATGGC**GGG**	-253 to -230	Antisense	62	Carnitine metabolism
CCA1 / YER168C	**GGG**TGTAT**GGG**C**GGGGG**AAATA**GGG**	-246 to -221	Sense	70	tRNA 3'-terminal CCA addition
COQ21/ YBR230W-A	**GGG**TAGTA**GGG**CA**GGG**AAA**GGG**	-154 to -132	Sense	69	N.D.
CUE4 / YML101C	**GGG**AGATAGGAA**GGGG**C**GGGGG**TCC**GGG**	-319 to -293	Antisense	66	N.D.
ECM3 / YOR092W	**GGGG**CGT**GGGG**CGT**GGG**ATATCGAAA**GGG**	-517 to -488	Antisense	67	N.D.
FIT2 / YOR382W	**GGG**TCCGCTGC**GGGG**CGA**GGGGGGG**	-245 to -220	Antisense	65	Siderophore transportation
GET2 / YER083C	**GGG**A**GGG**A**GGG**AGAAGTTT**GGG**	-196 to -174	Sense	65	Mitophagy, protein insertion into ER, retrograde vesicle-

					mediated transport
GPD1 / YDL022W	**GGG**ACTTT**GGG**TGCCC**GGGG**TGT**GGG**	-387 to -361	Antisense	71	Glycerol synthesis
HSP150 / YJL159W	**GGG**TCGCCTAATT**GGG**CAGAA**GGGGGG**	-499 to -472	Antisense	62	Cell-wall organization
HXK1 / YFR053C	**GGGG**CACACGTGC**GGG**AGTTTCAAA**GGGG**CAGAATAGT**GGGG**	-492 to -450	Antisense	72	Glucose metabolism
MEO1/ YBR126W-A	**GGG**CAGGTCA**GGG**CA**GGGG**CTCTCA**GGG**	-222 to -194	Antisense	68	N.D.
MRPL15 / YLR312W-A	**GGG**CACACC**GGGG**TA**GGGGG**TCCGGA**GGG**	-351 to -320	Sense	71	Mitochondrial ribosome subunit
MSN4 / YKL062W	**GGGG**A**GGGG**AAAAGAAAA**GGGG**AA**GGGG**	-897 to -869	Sense	100	Stress response transcription factor
PUN1 / YLR414C	**GGG**AGTGGAGA**GGG**CTCTGA**GGG**TCAAGT**GGG**	-413 to -381	Antisense	70	Cell-wall organization
RAD54 / YGL163C	**GGG**TTCCTTC**GGG**AGGA**GGGGGG**A**GGGG**	-895 to -873	Sense	69	Chromatin remodeling, DNA geometric change
RPH1 / YER169W	**GGG**TGTAT**GGG**C**GGGGG**AAATA**GGG**	-479 to -453	Antisense	70	Histone demethylase, transcription factor
RPL42B / YHR141C	**GGG**ATGAT**GGG**TTCCGCCA**GGGGG**A**GGG**	-178 to -150	Sense	67	Large ribosomal subunit
RPS18A / YDR450W	**GGG**AT**GGG**CATAT**GGG**ACT**GGG**	-192 to -170	Antisense	69	Small ribosomal subunit
RPS23A / YGR118W	**GGG**TAT**GGG**TGTATATA**GGG**TAT**GGG**	-394 to -369	Antisense	67	Small ribosomal subunit

RRN11 / YML043C	**GGG**C**GGGGGG**AAGTGATGGC**GGG**	-790 to -767	Sense	62	Component of rDNA transcription factor complex
RSC6 / YCR052W	**GGG**CCA**GGG**CTCCATGGC**GGG**TTCC**GGG**	-313 to -285	Sense	66	Subunit of RSC chromatin remodeling complex
SOD1/ YJR104C	**GGGG**T**GGG**TGCCCCTAA**GGG**AGC**GGG**	-209 to -182	Antisense	65	Regulation of transcription in response to oxidative stress
SPG4 / YMR107W	**GGG**AGATAAGA**GGG**AGCA**GGG**T**GGGG**	-185 to -159	Sense	66	N.D.
SUT1 / YGL162W	**GGG**TTCCTTC**GGG**AGGA**GGGGGG**A**GGGG**	-868 to -840	Antisense	69	Sterol uptake regulation
TPS1 / YBR126C	**GGG**CAGGTCA**GGG**CA**GGGG**CTCTCA**GGGGGG**	-262 to -233	Sense	68	Trehalose synthesis
TRM3 / YDL112W	**GGG**CACAAT**GGG**TGAGTTGTC**GGG**TAA**GGG**	-220 to -190	Sense	66	tRNA methylation
TSL1 / YML100W	**GGG**AGATAGGAA**GGGG**C**GGGGG**TCC**GGG**	-243 to -215	Sense	66	Trehalose synthesis
YCR047W-A	**GGG**CACC**GGG**ATACCGTTCC**GGG**C**GGG**	-81 to -55	Antisense	63	N.D.
YFL063W	**GGG**TTGGTGGT**GGGG**AGGCA**GGG**TAACGAGT**GGG**	-169 to -135	Antisense	70	N.D.
YGR117C	**GGG**TAT**GGG**TGTATATA**GGG**TAT**GGG**	-136 to -110	Sense	67	N.D.
YTM1 / YOR272W	**GGG**ATTC**GGG**CAAATCGAT**GGG**CCCT**GGG**	-813 to -785	Sense	67	Nucleolar protein subunit of PeBoW complex

Table S6. List of transcription factors that have guanine run in their consensus binding site.

Transcription factors	Consensus binding site	Classification
**Adr1**	GC**GGGG**	Zn-finger
Aft1	T**GGG**TGCA	N.D.
Aft2	**GGG**TGCG	N.D.
**Com2**	TCA**GGGG**T	Zn-finger
Crz1	**GGGG**CTG	Zn-finger
Flo8	C**GGGG**TTTTCT	N.D.
**Gis1**	TWA**GGG**AT	Zn-finger
Haa1	GGCGA**GGGG**	Copper-fist
Hot1	**GGG**ACAAA	N.D.
**Mig1**	ATTTTGC**GGGG**	Zn-finger
**Mig2**	ATTTTGC**GGGG**	Zn-finger
**Mig3**	ATTTTGC**GGGG**	Zn-finger
**Msn2**	A**GGGG**	Zn-finger
**Msn4**	A**GGGG**	Zn-finger
Nhp10	GCC**GGGG**A	HMG-box
Nrg1	A**GGGG**TCC	Zn-finger
Nrg2	MA**GGG**TCC	Zn-finger
Put3	CCC**GGG**	Zn-finger
Rap1	**GGG**TGTACGG	Myb-like
Rds2	TATC**GGG**	Zn-Finger
Reb1	CC**GGG**TAA	Myb-like
**Rgm1**	A**GGGG**	Zn-finger
**Rph1**	A**GGGG**	Zn-finger
**Rsf2**	AGC**GGGG**TA	Zn-finger
Sdd4	GC**GGGG**	Zn-finger
Stb4	TC**GGG**CCGA	Zn-finger
**Tda9**	GA**GGGG**	Zn-finger
**Uga3**	GGC**GGG**A	Zn-finger
Ume6	T**GGG**TGGCTA	Zn-finger
Usv1	A**GGGG**	Zn-finger
**YGR067c**	GC**GGGG**	Zn-finger

Consensus binding sites were extracted from published databases (6, 7). The protein names in bold are transcription factors enriched in stress response (8).

Table S7. Categories of genes that have G4 motif overlapping with STRE site

Category	Genes
Autophagy	*ATG20, ATG39*
Cellular enzyme	*ADO1, HXK1, RAD54, TRM3*
Membrane protein	*FIT2, PUN1*
Mitochondrial protein	*COQ21, MRPL15*
Ribosomal protein	*RPL42B*
Stress response	*HSP150, SOD1*
Transcription factor	*MSN4, SUT1*
Trehalose synthesis	*TPS1, TSL1*
Unknown function	*CUE4, ECM3, MEO1*

Materials and Methods for Supplemental Data.

Circular Dichroism analyses.

For the oligos indicated in Figure S1, 10 μM solutions containing 100 mM K^+ were first heated to 95°C for 5 mins and slowly cooled to room temperature before the CD analyses, which were carried out by Creative Proteomics (Shirley, NY). 200 μL of each DNA sample was loaded on Chirascan circular dichroism spectrometer and scanned from 190 to 320 nM. Each scan was carried out at room temperature with the path length of 1 mm and the scan speed of 0.5 sec/point. Each sample was scanned for 3 repeats and the spectra were generated by using the average value of 3 repeats.

Legends for Supplemental Figures.

Figure S1. Circular Dichroism analyses of G4-forming oligos.

Chirascan circular dichroism spectrometer was used to scan 10 μM solutions of the indicated oligos containing 100 mM K^+ at room temperature with the path length of 1mm and the scan speed of 0.5 sec/point. The sequences of oligos are same as those listed in Figures 1F and 3A. CD analyses were carried out by Creative Proteomics (Shirley, NY).

Figure S2. Purification of GST-Msn2(596-704) zinc finger domain.

The GST-Msn2(596-704) was expressed in *E. coli* and purified using glutathione agarose gel. The purification samples were run on SDS-PAGE gel followed by Coomassie staining. W1, W2, W3: Sequential washes. E1, E2, E3: Sequential elution fractions.

Figure S3. Purified GST-Msn2(596-704) binds to STRE-containing *dsCTT1* oligos.

A) Sequences of *dsCTT1* and *dsCTT1STREMut* are shown. Oligos were annealed from complementary single strand oligos in buffers containing either 100 mM K^+ or 100 mM Li^+ as indicated and then subjected to EMSA with GST-Msn2(596-704). B) GST-Msn2(596-704) bound selectively to *dsCTT1* but not *dsCTT1STREMut.* C) The binding of GST-Msn2(596-704) to *dsCTT1* in K^+ buffer was similar to that in Li^+ buffer. B) and C) Bands representing free or Msn2(596-704)-bound oligos are indicated.

Figure S4. Determining the Kd of GST-Msn2(596-704) binding to *ATG39pG4* oligo.

A) *ATG39pG4* oligo was folded in 100 mM K^+ and subjected to EMSA with indicated concentrations of GST-Msn2(596-704) protein. 1 pmol of *ATG39pG4* oligo was used for each reaction. Unbound G4 DNA and G4-Msn2(596-704) bands are indicated. B) EMSA-derived saturation binding curve of GST-Msn2(596-704) to *ATG39pG4*. Experiments were repeated three times and error bars indicating standard deviations are shown on the graph.

Figure S5. G4 ligand treatment induces *FET3* mRNA level.

The mid-log phase WT cells were treated with G4 ligand for 2 generations and total RNAs were extracted for RT-qPCR analysis with sample size n = 4 biological replicates. The significant difference was determined by Student's *t*-test with (**) p-value < 0.01.

Figure S6. Deletion of *MSN2/4* reduces transcription induction caused by PhenDC3.

Total RNAs were extracted for RT-qPCR analysis from the mid-log phase WT and *msn2*Δ *msn4*Δ cells treated with 25 μM PhenDC3 for 2 generations. qRT-PCR was used to measure the transcript level of each indicated genes. Primer sequences are listed in Table S2. Sample size N = 4 biological replicates. The significant difference was determined by Student's *t*-test. **; p-value < 0.01, ***; p-value < 0.001, and ****; p-value < 0.0001. The graphs show mRNA fold changes relative to that of untreated WT sample. (A) *ATG20*, (B) *ATG39*, (C) *TPS1*, and (D) *TSL1*.

Figure S7. EMSA with *c-KIT2* oligos.

Sequences of *cKIT2 and cKIT2Long* are shown in the box. Oligos were annealed in buffers containing either 100 mM K^+ or 100 mM Li^+ as indicated and then subjected to EMSA with GST-Msn2(596-704).

Supplemental References

1. Sikorski RS, Hieter P. A system of shuttle vectors and yeast host strains designed for efficient manipulation of DNA in Saccharomyces cerevisiae. Genetics. 1989;122(1):19-27.
2. Kim N, Jinks-Robertson S. Guanine repeat-containing sequences confer transcription-dependent instability in an orientation-specific manner in yeast. DNA Repair (Amst). 2011;10(9):953-60.
3. Lopez CR, Singh S, Hambarde S, Griffin WC, Gao J, Chib S, et al. Yeast Sub1 and human PC4 are G-quadruplex binding proteins that suppress genome instability at co-transcriptionally formed G4 DNA. Nucleic Acids Res. 2017;45(10):5850-62.
4. Gueldener U, Heinisch J, Koehler GJ, Voss D, Hegemann JH. A second set of loxP marker cassettes for Cre-mediated multiple gene knockouts in budding yeast. Nucleic Acids Res. 2002;30(6):e23.
5. Longtine MS, McKenzie A, 3rd, Demarini DJ, Shah NG, Wach A, Brachat A, et al. Additional modules for versatile and economical PCR-based gene deletion and modification in Saccharomyces cerevisiae. Yeast. 1998;14(10):953-61.
6. Teixeira MC, Monteiro PT, Palma M, Costa C, Godinho CP, Pais P, et al. YEASTRACT: an upgraded database for the analysis of transcription regulatory networks in Saccharomyces cerevisiae. Nucleic Acids Res. 2018;46(D1):D348-D53.
7. Gordan R, Murphy KF, McCord RP, Zhu C, Vedenko A, Bulyk ML. Curated collection of yeast transcription factor DNA binding specificity data reveals novel structural and gene regulatory insights. Genome Biol. 2011;12(12):R125.
8. Kuang Z, Pinglay S, Ji H, Boeke JD. Msn2/4 regulate expression of glycolytic enzymes and control transition from quiescence to growth. Elife. 2017;6.

Figure S1

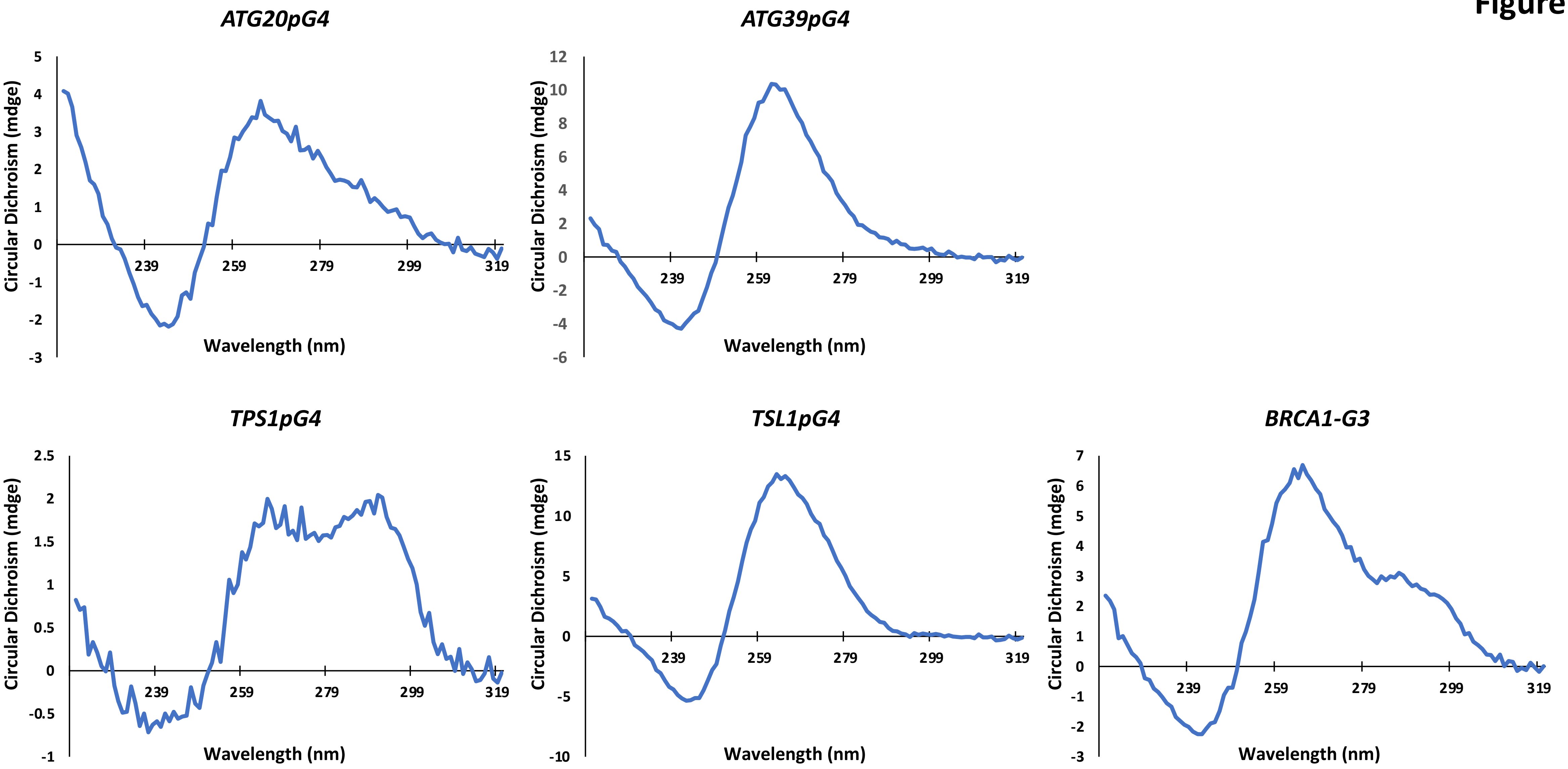
ATG20pG4
ATG39pG4
TPS1pG4
TSL1pG4
BRCA1-G3
Circular Dichroism (mdge)
Wavelength (nm)
239
259
279
299
319
5
4
3
2
1
0
-1
-2
-3
12
10
8
6
4
2
0
-2
-4
-6
2.5
2
1.5
1
0.5
0
-0.5
-1
15
10
5
0
-5
-10
7
6
5
4
3
2
1
0
-1
-2
-3

Figure S2

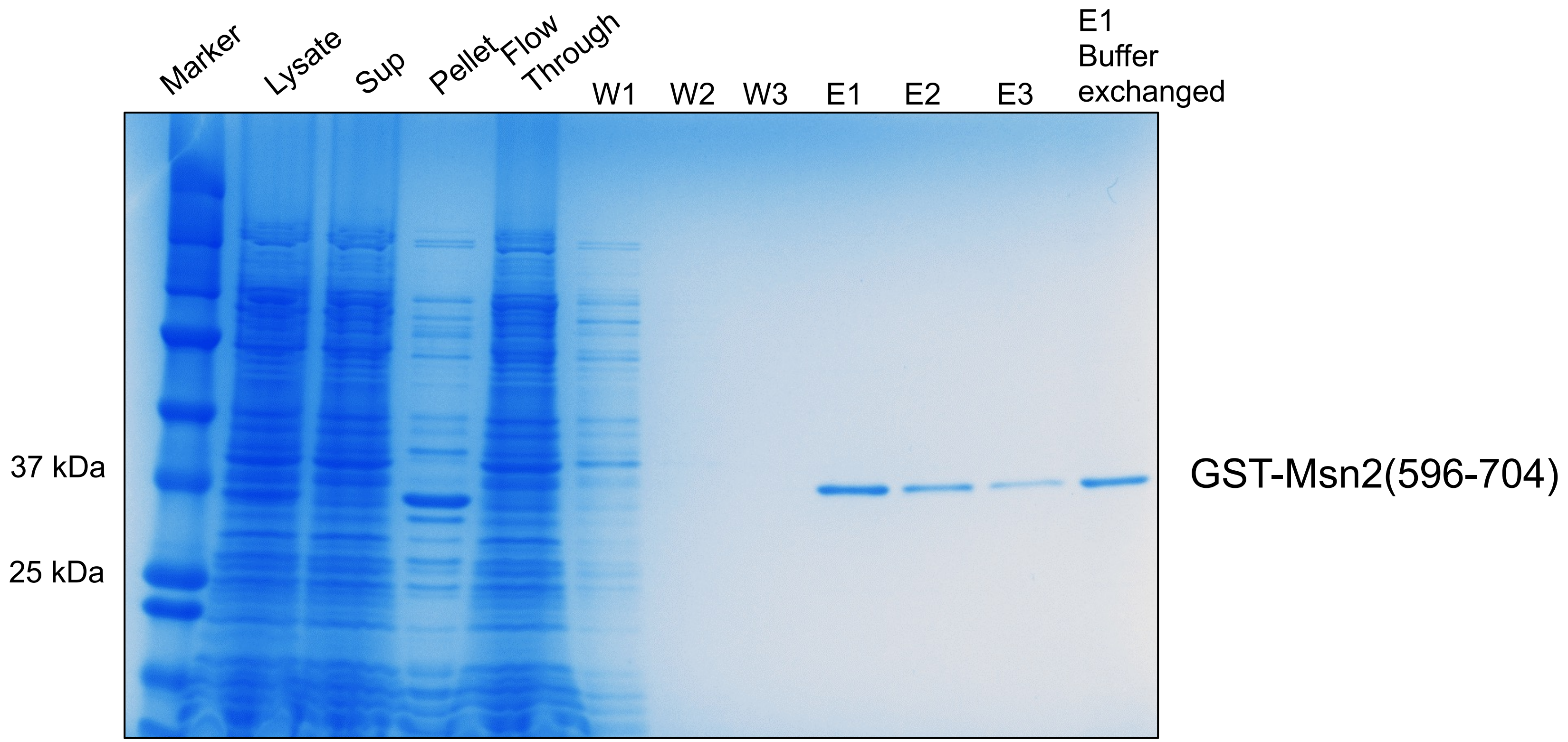

Marker
Lysate
Sup
Pellet
Flow Through
W1
W2
W3
E1
E2
E3
E1 Buffer exchanged
37 kDa
25 kDa
GST-Msn2(596-704)

Figure S3

A.

dsCTT1

5′CTCTTTTTCA**AGGGG**ATCACCGGTA**AGGGG**CCAATCTGTG
3′GAGAAAAAGT**TCCCC**TAGTGGCCAT**TCCCC**GGTTAGACAC

dsCTT1STREMut

5′CTCTTTTTCA**TGTGT**ATCACCGGTA**TGTGT**CCAATCTGTG
3′GAGAAAAAGT**ACACA**TAGTGGCCAT**ACACA**GGTTAGACAC

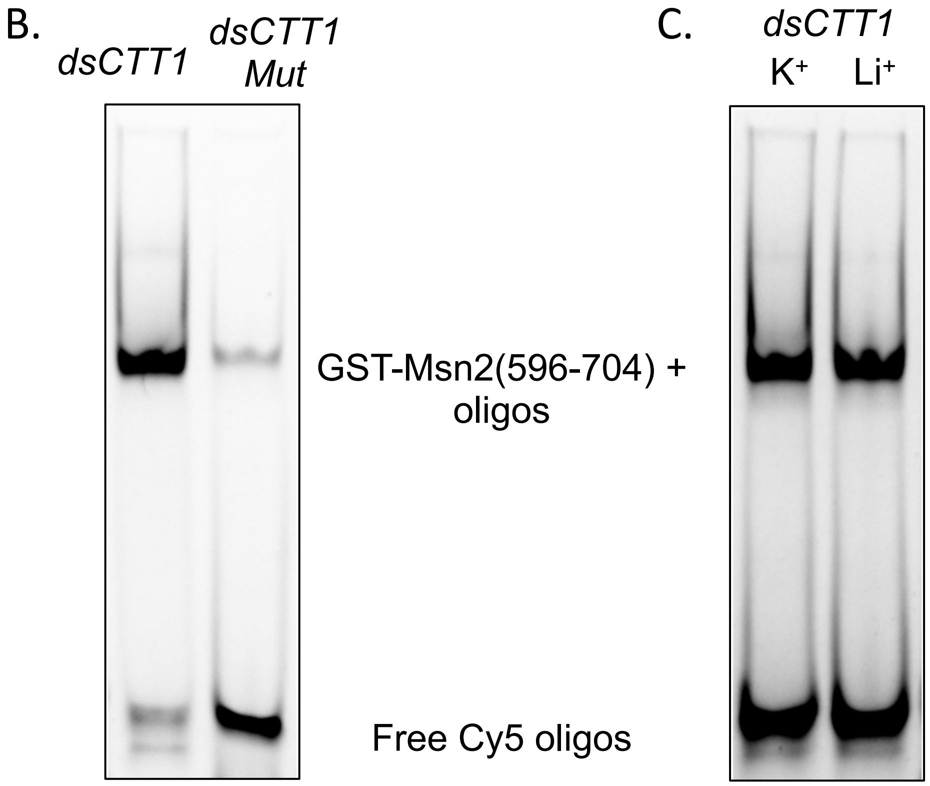

Figure S4

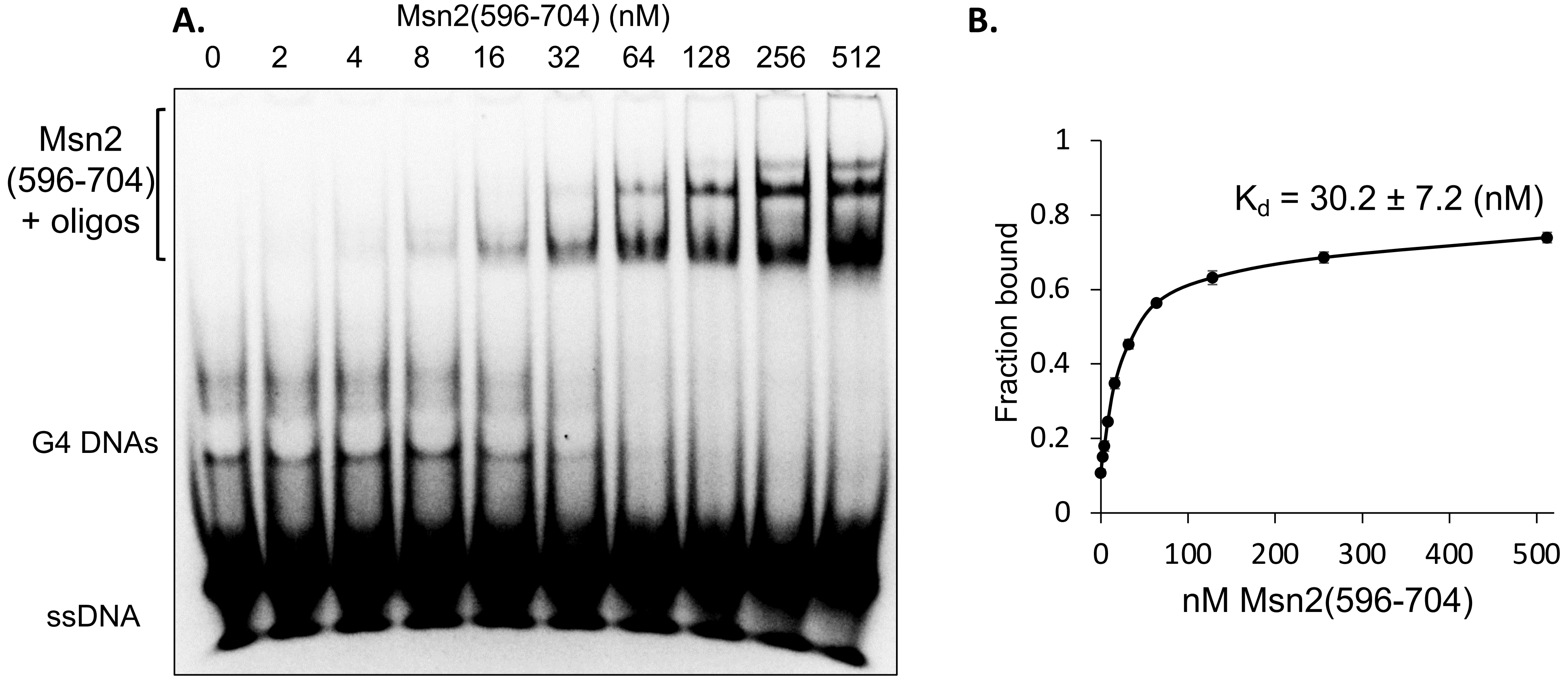
A.
Msn2(596-704) (nM)
0 2 4 8 16 32 64 128 256 512
Msn2
(596-704)
+ oligos
G4 DNAs
ssDNA
B.
K$_d$ = 30.2 ± 7.2 (nM)
Fraction bound
0
0.2
0.4
0.6
0.8
1
0 100 200 300 400 500
nM Msn2(596-704)

Figure S5

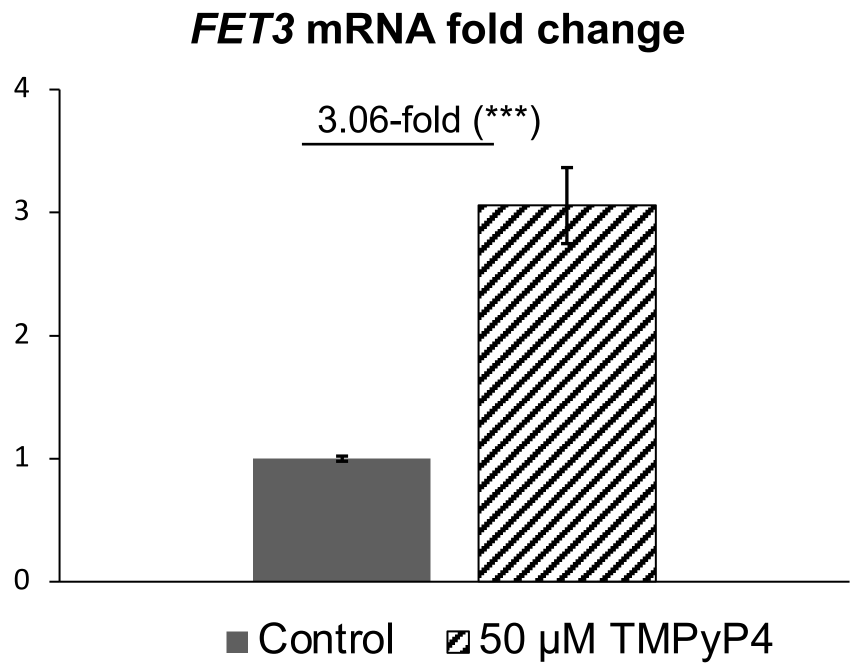

Figure S6

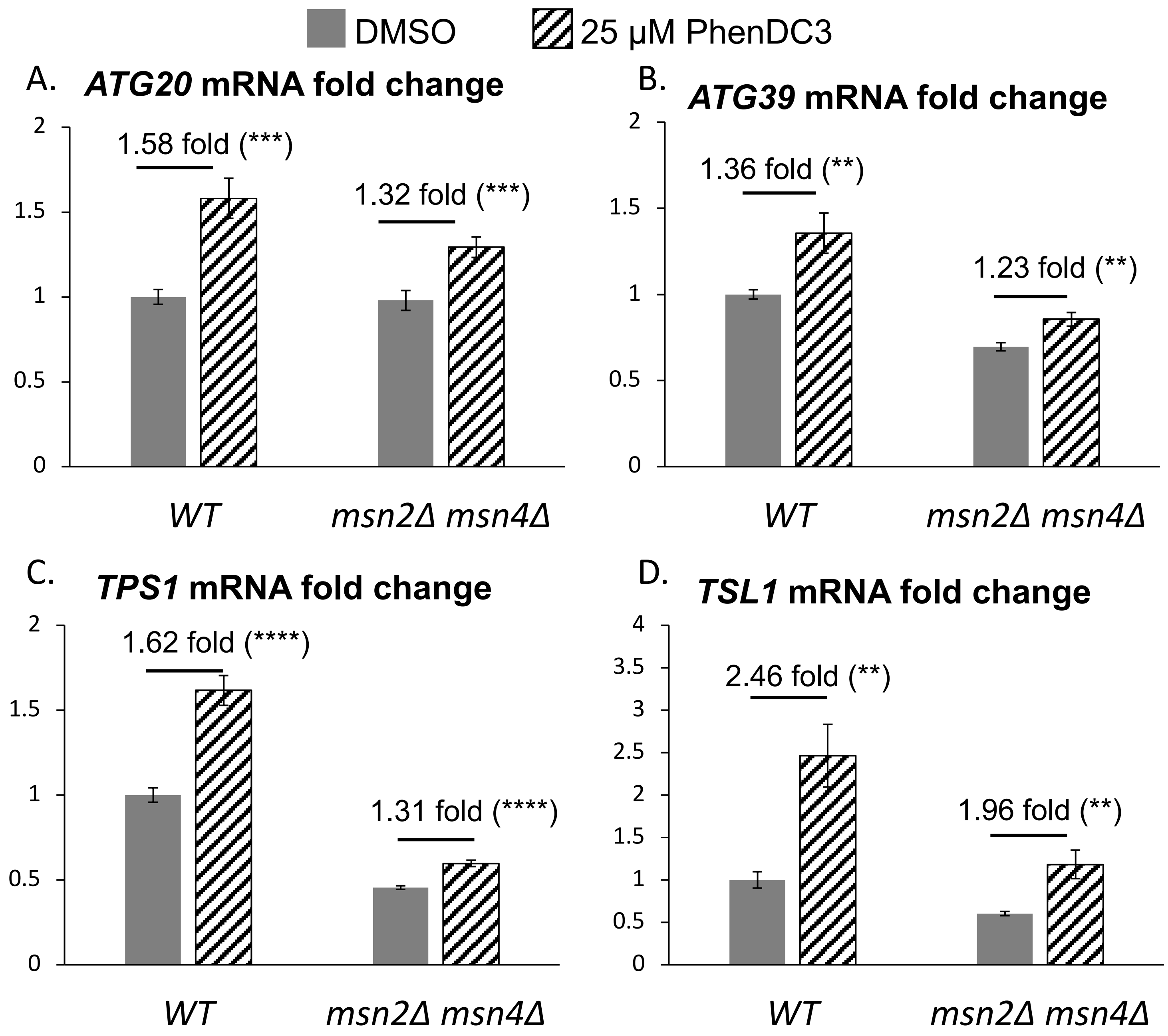
DMSO
25 µM PhenDC3
A.
ATG20 mRNA fold change
2
1.5
1
0.5
0
1.58 fold (***)
1.32 fold (***)
WT
msn2Δ msn4Δ
B.
ATG39 mRNA fold change
2
1.5
1
0.5
0
1.36 fold (**)
1.23 fold (**)
WT
msn2Δ msn4Δ
C.
TPS1 mRNA fold change
2
1.5
1
0.5
0
1.62 fold (****)
1.31 fold (****)
WT
msn2Δ msn4Δ
D.
TSL1 mRNA fold change
4
3.5
3
2.5
2
1.5
1
0.5
0
2.46 fold (**)
1.96 fold (**)
WT
msn2Δ msn4Δ

Figure S7

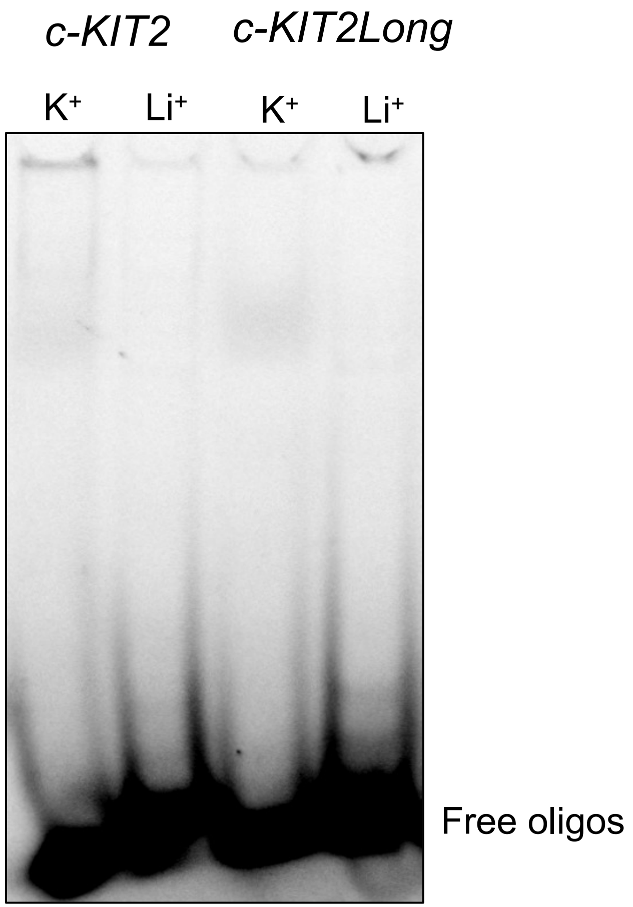

c-KIT2	**GGG**C**GGG**CGCGA**GGG**A**GGGG**
c-KIT2Long	CCC**GGG**C**GGG**CGCGA**GGG**A**GGGG**AGG
